# Language production impairments in patients with a first episode of psychosis

**DOI:** 10.1371/journal.pone.0272873

**Published:** 2022-08-11

**Authors:** Giulia Gargano, Elisabetta Caletti, Cinzia Perlini, Nunzio Turtulici, Marcella Bellani, Carolina Bonivento, Marco Garzitto, Francesca Marzia Siri, Chiara Longo, Chiara Bonetto, Doriana Cristofalo, Paolo Scocco, Enrico Semrov, Antonio Preti, Lorenza Lazzarotto, Francesco Gardellin, Antonio Lasalvia, Mirella Ruggeri, Andrea Marini, Paolo Brambilla, GET UP Group

**Affiliations:** 1 Department of Pathophysiology and Transplantation, University of Milan, Milan, Italy; 2 Department of Neurosciences and Mental Health, Fondazione IRCCS Ca’ Granda-Ospedale Maggiore Policlinico, Milan, Italy; 3 Department of Neuroscience, Biomedicine and Movement Sciences, University of Verona, Verona, Italy; 4 Verona Hospital Trust–Azienda Ospedaliera Universitaria Integrata Verona–AOUI, Verona, Italy; 5 IRCCS “E.Medea” Polo Friuli Venezia Giulia, San Vito al Tagliamento, PN, Italy; 6 Department of Languages and Literatures, Communication, Education and Society, University of Udine, Udine, Italy; 7 Department of Mental Health, Azienda ULSS 16, Padua, Italy; 8 Department of Mental Health, Reggio Emilia, Italy; 9 Department of Mental Health, Niguarda Ca’ Granda Hospital, Milan, Italy; 10 Department of Mental Health, Azienda Ulss 8 Berica, Vicenza, Italy; University of California Davis, UNITED STATES

## Abstract

Language production has often been described as impaired in psychiatric diseases such as in psychosis. Nevertheless, little is known about the characteristics of linguistic difficulties and their relation with other cognitive domains in patients with a first episode of psychosis (FEP), either affective or non-affective. To deepen our comprehension of linguistic profile in FEP, 133 patients with FEP (95 non-affective, FEP-NA; 38 affective, FEP-A) and 133 healthy controls (HC) were assessed with a narrative discourse task. Speech samples were systematically analyzed with a well-established multilevel procedure investigating both micro- (lexicon, morphology, syntax) and macro-linguistic (discourse coherence, pragmatics) levels of linguistic processing. Executive functioning and IQ were also evaluated. Both linguistic and neuropsychological measures were secondarily implemented with a machine learning approach in order to explore their predictive accuracy in classifying participants as FEP or HC. Compared to HC, FEP patients showed language production difficulty at both micro- and macro-linguistic levels. As for the former, FEP produced shorter and simpler sentences and fewer words per minute, along with a reduced number of lexical fillers, compared to HC. At the macro-linguistic level, FEP performance was impaired in local coherence, which was paired with a higher percentage of utterances with semantic errors. Linguistic measures were not correlated with any neuropsychological variables. No significant differences emerged between FEP-NA and FEP-A (p≥0.02, after Bonferroni correction). Machine learning analysis showed an accuracy of group prediction of 76.36% using language features only, with semantic variables being the most impactful. Such a percentage was enhanced when paired with clinical and neuropsychological variables. Results confirm the presence of language production deficits already at the first episode of the illness, being such impairment not related to other cognitive domains. The high accuracy obtained by the linguistic set of features in classifying groups support the use of machine learning methods in neuroscience investigations.

## Introduction

Language is one of the most complex human skills, resulting from the dynamic integration of processes that require the combined functioning of several brain areas. Given the fundamental importance that language has in characterizing human beings and as a primary source for clinicians to assess both strengths and weaknesses in patients, its study in mental illness, and especially in psychosis, is particularly interesting. Patients with psychosis usually have difficulties in selecting contextually appropriate words, and often fill their speech with irrelevant pieces of information and derailments [1]. Impairments are generally observed in a range of psychotic disorders, including schizophrenia and bipolar disorder [2, 3]. In the prodromal phase of the illness, some language production deficits are even considered to be specific predictors of schizophrenia subtypes, and their presence influences the prognosis [4–6]. A fascinating theory by Crow has even hypothesized a central role of language in the development of schizophrenia [7]. According to such hypothesis, psychotic symptoms may be related to an altered hemispheric lateralization [8], with language lateralization during language production tasks being significantly reduced in psychotic patients even at the onset of the symptoms [9, 10]. Language processing rests on the integration between different systems (e.g., those processing phonological, morphological, syntactic, semantic or pragmatic information, see [11, 12], for reviews), so their systematic investigation can represent a useful tool to clarify the nature of the linguistic deficits observed in psychosis and to guide the understanding of the pathogenic trajectories of the disease from first episodes to the chronic condition. While the current literature on language impairments is quite large for psychotic patients, also including several papers by our group [1, 12–17], with chronic patients showing alterations in both language comprehension and production [18], much less emphasis has been given to the first episode so far. In previous studies, linguistic impairments have been observed in patients with a first episode of psychosis (FEP) at the receptive level [15–17, 19, 20]. By contrast, the investigation of the linguistic productive level has been less investigated in the first phases of the illness. For example, some studies on patients with a clinical high risk (CHR) to develop psychosis show that language production variables, particularly at the syntactic and semantic level of discourse, can be predictive of psychosis onset [21, 22]. Despite that, very few studies evaluate speech production in FEP in a detailed and systematic way. Existing studies usually focus on relating the patients’ outcomes and prognosis to various clinically objectifiable deficits, including some in the language department [23]. Also, most of the available studies on linguistic production skills in FEP use clinical scales to assess language, thus producing confusing results as they do not always take into account the difference between thought and language variables, therefore missing out on important areas of language production such as discourse, cohesion, naming abstraction and semiotics [24, 25]. Ayer and colleagues [26], for example, used the Thought and Language Index (TLI) [24] to evaluate formal thought disorders (FTD) in FEP patients, showing poverty of speech, perseveration and peculiar word use as significant factors in differentiating FEP from HC. Other studies combine clinical findings and a natural language processing (NLP) approach, consisting in a language analysis made by an artificial intelligence which is programmed to understand text and spoken words in a similar way to human beings [27, 28]. Silva et al, 2021 [29], for example, found that people with a first episode of schizophrenia (FES) have a linguistic style characterized by reduced analytic thinking, with less categorical linguistic style (that is with less formal and hierarchical patterns) than healthy controls (HC), but using the same proportion of function (including articles, prepositions, conjunctions, non-referential adverbs, negations, and auxiliary verbs) and content words (words that hold meaning on their own). Disorganization as a clinical item also seems to be related to an aberrant use of connectives (specifically increased temporal but decreased use of causal connectives) [30].

Despite recent findings that have proven new insight on linguistic production deficits in FEP, an overall comprehension of language production impairments in this population is still missing. For this reason, the first aim of the present study is to investigate linguistic production abilities in a large sample of FEP, according to a well-established and systematic methodology that has been specifically designed to evaluate language production in all its sub-domains and variables [31]. In particular, we focused on both micro- (lexicon, morphology, syntax) and macro-linguistic (discourse coherence, pragmatics) dimensions of language [31].

Another interesting issue in clinical research is represented by the investigation of similarities and differences in patients with affective and non-affective psychosis. In previous studies by our group, we detected linguistic deficits in both affective and non-affective chronic patients at both receptive [13, 15–17] and productive levels [14], with more severe and generalized impairment in patients with schizophrenia than in those with bipolar disorder. In particular, in narrative production, participants with schizophrenia had slight problems in speech rate (that is number of word/unit time) and deficits at both local and global discourse coherence, whereas patients with bipolar disorder showed reduced mean length of utterance, compared with healthy participants [14]. Other studies show that patients with bipolar disorder usually display poverty of speech and content, as well as circumstantiality and self-reference. These findings, however, usually concern patients in the depressive state of the illness, aside from circumstantiality, which can be portrayed by patients in the manic phase as well [32]. Manic patients also usually are known to make clang associations (those associations based on similarities of sounds). An interesting study by Docherty et al [33] comparing schizophrenia and bipolar disorder patients shows that schizophrenic patients produce utterances with a relevantly lower syntactic complexity than patients with mania, even at an early stage of illness. Despite some type of language production impairment has been observed in both affective and non-affective chronic patients, specific deficits have not been clearly identified in a conclusive manner in FEP. A study by Kravariti et al (2009) showed FEP patients with positive history of mania to have an isolated, selective deficit in semantic verbal fluency [34], while another [23] identified verbosity as the only FTD category which could discriminate between FEP patients with a mania or schizophrenia diagnosis. Again, however, these results are derived from analysis on clinical scales that do not take into account the full complexity of natural language. Given such a premise, the second aim of the present study is to compare affective and non-affective FEP patients in an effort to compare their language production profiles with the aim of improving our comprehension of these pathologies [35]. A further intriguing issue concerns the relationship between language and other cognitive skills. In chronic patients with psychosis different cognitive abilities are altered (e.g., language, memory, attention) [36–38]. As these complex functions strongly affect one another, it is not clear yet whether language deficits are to be considered at least partly independent of other cognitive dysfunctions [e.g., 39–41]. The potential relation between different cognitive difficulties may be easier to evaluate in persons with FEP, where dysfunctions are generally quite moderate and the effects of medication and chronicity are not prevalent.

In sum, the present study aimed to compare language production abilities in a large cohort of patients with FEP compared to a group of HC, by considering both affective (FEP-A) and non-affective (FEP-NA) and taking into account the potential interrelations between different cognitive impairments. In addition to classical statistical analyses, we also adopted a machine learning (ML) approach in order to test the predictive values of the linguistic variables in discriminating between FEP vs HC and FEP-NA vs FEP-A. Specifically, we hypothesized that a) patients with FEP would show deficits in linguistic performance compared to HCs; b) patients with FEP-NA would display different profiles compared to FEP-A; c) some language difficulties would be to some extent independent from other cognitive impairments while others (e.g., language planning and discourse organization) would be more related to other cognitive skills (e.g., executive functions), and d) linguistic production variables can discriminate between FEP and HC and, possibly, between FEP-NA and FEP-A.

## Materials and methods

### Participants

Three-hundred thirty-nine Italian speaking adults with no history of drug or alcohol abuse and with normal or corrected to normal vision and hearing took part in this study. They formed an experimental and a control group. The experimental group was formed by 206 outpatients with FEP that were recruited from 117 public community mental health centers (CMHC) located in the north of Italy in the frame of the study ‘Genetics Endophenotypes and Treatment: Understanding early Psychosis’ (the GET UP study; [42]). The GET UP inclusion criteria were: age 18–54 years, residence in the catchment regions of the CMHCs and first lifetime contact, presence of at least one of the following symptoms: hallucinations, delusions, qualitative speech disorder, qualitative psychomotor disorder, bizarre, or grossly inappropriate behavior, or two of the following: loss of interest, initiative, and drive; social withdrawal; episodic severe excitement; purposeless destructiveness; overwhelming fear; or marked self-neglect. Exclusion criteria were: antipsychotic treatment (>3 months) prescribed for an identical or similar mental disorder; mental disorders caused by a general medical condition; moderate or severe mental disability evaluated by a clinical functional assessment; and psychiatric diagnosis other than International Classification of Diseases (ICD)-10 [43] for psychosis. The specific ICD-10 codes for psychosis were assigned at 9 months. Diagnoses were made by using the Item Group Checklist (IGC) of the Schedule for Clinical Assessment in Neuropsychiatry (SCAN) [44] and were confirmed by the clinical consensus of two staff psychiatrists. Participants’ social, occupational, and psychological functioning were assessed with the Global Assessment of Functioning (GAF) scale [45], while FEP positive and negative symptoms were assessed by means of the Positive and Negative Syndrome Scale (PANSS) [46]. Patients were also assessed for the presence or absence of affective symptoms by means of the Hamilton Depression Rating Scale (HDRS) [47] and the Bech-Rafaelsen Mania Rating Scale (BRMRS) [48]. In addition, FEP mean duration (in days) of untreated psychosis (DUP), defined as the time from onset of first psychotic symptom (as reported by patients) to first contact with public mental health services, was recorded.

A group of 133 healthy participants was also recruited by word of mouth in the frame of other studies at the AOUI of Verona, Italy. Participants in the control group had no DSM-IV Axis I disorders, determined using a brief modified version of the Structured Clinical Interview for DSM-IV–Non-Patient Version, no history of psychiatric disorder among first-degree relatives and alcohol or substance misuse, no current major medical illness. Since a core part of the present study involves machine learning (ML) analyses, we randomly sampled the entire set of data of FEP and HC in order to obtain a final dataset of 133 FEP and 133 HC (Table 1). All the data, analyses and results described from here onward will be referred to this final dataset. According to the ICD-10 criteria, 38 out of 133 patients were classified as having affective psychosis, FEP-A (F30.2, F31.2, F31.5, F31.6, F32.3, F33.3), while 95 received a diagnosis of non-affective psychosis, FEP-NA (F20-F29) (see S1 Table for a description of FEP-NA and FEP-A).

**Table 1:** Sociodemographic and clinical data of the sample of FEP and HC.

	HC (n = 133)	FEP (n = 133)	Comparison
Female %	51.88%	39.85%	χ2(1) = 3.41, p = 0.07
Age in years	33.07 ±9.56 (19, 64)	28.93 ±9.05 (16, 53)	t(263.2) = 3.62, p<0.00*
Education in years	15.62 ±3.59 (5.0, 26.0)	11.58 ±3.06 (5.0, 18.0)	t(257.7) = 9.85, p<0.00*
GAF	83.14 ±6.19	46.31 ±12.94	t(197.3) = 28.64, p<0.00*
PANSS	-	34.95 ±8.93	-
HAM-D	-	15.23 ±7.875 (0.0, 61.0)	-
BRMRS	-	2.59 ± 3.40	-
DUP	-	294.87 ± 571.99	-

HC, Healthy Controls; FEP, First Episode Psychosis; GAF, Global Assessment of Functioning

PANSS, Positive and Negative Syndrome Scale, General psychopathology subscale; HAM-D, Hamilton’s Depression Rating Scale; BRMRS, Bech-Rafaelsen Mania Rating Scale; DUP, Duration of untreated psychosis.

The study was approved by the Ethics Committee of the AOUI of Verona (Prot. N. 20406/CE; N. 1877; N. 1338; N. 2290) and, only for patients, from the local Ethic Committee of each site of recruitment. All participants gave signed informed consent, after an explanation of all issues involved in participation in the research. The entire procedure and the informed consent form were drawn up according to the declaration of Helsinki [49] and approved by the Ethics Committee, which did not require further procedures to assess the capacity to consent.

### Linguistic production measures

The participants’ narrative production skills were assessed by adopting a multilevel and systematic procedure already published by our group [13, 14, 31, 50]. Patients with FEP and HC were asked to provide a verbal description of a drawing representing the story of a boy trying to reach a bird’s nest (The Nest Story; [51]). No time limit was given, and the evaluator was instructed to refrain from intervening. The narrative productions were recorded and later transcribed verbatim with the inclusion of phonological fillers, pauses, and false starts by two independent coders who had been previously trained. The transcripts were compared for ensuring intercoder reliability. The transcripts underwent an accurate manual linguistic analysis by the same coders, focusing on both micro- and macro-linguistic levels of processing, which refer respectively to the intra-phrasal (lexicon, morphology, syntax) and inter-phrasal (discourse coherence and pragmatics) levels of language processing [31]. An in-depth description of linguistic variables and their meaning is detailed in the S1 File.

### Neuropsychological assessment

All participants were administered tasks assessing global cognitive and executive functioning. In particular, verbal intelligence quotient (V-IQ) was assessed by administering the Test di Intelligenza Breve (TIB; [52]). Working memory and the ability to process multiple units of information by short term memory were assessed with the n-Back task (adapted from Kirchner, [53]) and the Span of Apprehension task, SOA (adapted from Asarnow and MacCrimmon, [54]), respectively. For a detailed description of the cognitive tasks, please refer to the S2 File.

### Statistical analyses

Analyses were performed using the SciPy library, version 1.8.0, for Python 3.10 (https://scipy.org). Continuous socio-demographic, clinical, neuropsychological and linguistic variables were entered into different series of t-tests for independent samples, comparing FEP and HCs. If the main effect was statistically significant (i.e. the comparison between FEP and HC gave a p≤0.05), post-hoc t-tests further explored the differences between FEP-NA and FEP-A, FEP-NA vs HC and FEP-A vs HC. For the post-hoc comparisons, the Bonferroni correction was applied adjusting the alpha level according to the number of comparisons to a value alpha = 0.02. The differences between groups in the distribution of males and females were analyzed by means of the Chi2 Fisher’s exact test. Pearson’s correlations were performed separately for the group of FEP and the group of HC in order to investigate the relationship between the linguistic and neuropsychological variables which resulted statistically significant in the t-tests (alpha level corrected for multiple comparisons).

### Machine learning (ML) analyses

A machine learning (ML) algorithm including independent variables (socio-demographic, neuropsychological and linguistic measures) was used to classify the two diagnostic groups (FEP and HC). The eXtreme Gradient Boosting algorithm was used. This is an improved version of decision trees first proposed by Chen & Carlos Guestrin [55] which uses a gradient optimization technique to better fit several decision trees to the training data, which then contribute to give a global more reliable response. To prevent overfitting of the models and more accurate results, we performed a train-test split of the dataset, the former used for training the model, the latter to measure accuracy of the trained model. We applied the common train/test splitting ratio of 80%/20%. We used the Julia language, version 1.6, and its ScikitLearn.jl package, a part of the ScikitLearn package available for Python mainly for data preprocessing and plotting; we also implied the XGBoost port of the XGBoost C++ algorithm implementation, version 1.4.0 [https://xgboost.readthedocs.io], for Python, version 3.10, called from the Julia environment. We tuned the main XGBoost parameter, max_depth, a hyperparameter setting the maximum depth of the trees in the model, with a grid search based on cross validation scores to select the best value. Maximum depth of the trees clearly improves model complexity so that its tuning is necessary to balance and set the model between underfitting and overfitting. Due to the expected potential variations of the predictive models’ accuracy, we repeatedly simulated fitting experiments to address these variations. For this purpose, the model has been fitted several times, randomly splitting at each run the dataset in train and test cases for 100 runs. We first fitted and tested our model choosing as predictors language production features only. Then, in order to improve the model’s discriminative power, we fitted and tested further models by adding language production variables to models trained on sociodemographic and neuropsychological data only. As a reference threshold, we list the "dummy" mode classifier accuracy, which always predicts the most represented class. Finally, in the model containing only the linguistic measures, we extracted feature importance in order to establish which linguistic production variables are the most useful in differentiating FEP and HCs and their relative contribution to the model’s accuracy.

## Results

### Multilevel assessment of narrative language

Patients with FEP as a group obtained worse performance than HC in measures assessing both the microlinguistic and macrolinguistic domains.

At micro-linguistic level, FEP and HC reported significantly worse Speech Rate, Mean Length of Utterances, Phonological Paraphasias, Lexical Fillers and Syntactic Completeness (all p<0.05) (Table 2).

**Table 2:** Linguistic variables in HC and FEP.

	HC (N = 133)	FEP (N = 133)	
Microlinguistic measures	Mean ± st.dev.	Mean ± st.dev.	Comparison
False-starts	0.94 ±1.14	1.19±2.91	t(171.6) = -0.92, p = 0.361
Speech-rate (words per minute)	134.29 ±25.31	119.43 ±38.37	t(228.6) = 3.73, p<**0.001**
Mean length of utterances (words)	7.19±1.67	6.23 ±1.53	t(261.8) = 4.86, p<**0.001**
Phonological paraphasias	0.05±0.26	0.14 ±0.40	t(223.1) = -1.99, p = **0.047**
Semantic paraphasias	0.19±0.64	0.35±0.75	t(257.9) = -1.85, p = 0.066
Verbal paraphasias	0.04±0.19	0.10±0.32	t(214.4) = -1.85, p = 0.066
Undefined words	0.00±0.00	0.02±0.15	t(132.0) = -1.75, p = 0.083
Lexical fillers	7.02±8.97	4.74 ±7.61	t(257.1) = 2.23, p = **0.026**
Syntactic completeness (%)	45.90±18.91	40.13 ±21.71	t(259.1) = 2.31, p = **0.022**
**Macrolinguistic measures**			
Local coherence errors (ambiguous)	0.34±0.61	0.53 ±0.83	t(243.1) = -2.18, p = **0.030**
Local coherence errors (missings)	1.25±1.36	0.93 ±1.04	t(246.7) = 2.13, p = **0.034**
Utterances with semantic errors (words)	1.58±3.52	3.35 ±8.84	t(172.8) = -2.14, p = **0.034**
Repetitions (%)	5.08±4.00	6.20±5.26	t(246.5) = -1.96, p = **0.052**
Global coherence errors (%)	0.00±0.00	0.06±0.47	t(132.0) = -1.40, p = 0.165

HC, Healthy Controls; FEP, First Episode Psychosis.

Post-hoc comparisons showed that FEP-NA did significantly worse than HC for Speech Rate, Mean Length of Utterances, and Syntactic Completeness (all p<0.02), while the difference between FEP-NA and HC for their production of Lexical Fillers did not survive the Bonferroni correction (p>0.02). At a macro-linguistic level, FEP did more Local Coherence Errors and produced more Utterances with Semantic Errors than HC (all p<0.05). Post-hoc comparisons showed that only the difference between FEP-NA and HC for the production of Utterances with Semantic Errors and the difference between FEP-A and HC for the Local Coherence Errors (missing) passed the Bonferroni correction threshold (p<0.02), suggesting that both FEP-NA and FEP-A contributed equally in the variability of the measures of Local Coherence Errors (ambiguous) and the production of Utterances with Semantic Errors (p>0.02). See Tables 3–5 for details.

**Table 3:** Post-Hoc analysis (HC vs FEP-NA).

	HC (N = 133)	FEP-NA (N = 95)			
Microlinguistic measures	Mean ± st.dev.	Mean ± st.dev.	t-test	df	p-value
Speech-rate (words per minute)	134.29±25.31	119.56±40.62	-3.13	145.40	**0.00**
Mean length of utterances (words)	7.19 ±1.67	6.23±1.53	-4.42	212.12	**0.00**
Phonological paraphasias	0.05±0.26	0.16±0.42	2.17	143.08	0.03
Lexical fillers	7.02±8.97	4.63±7.91	-2.12	216.14	0.04
Syntactic completeness (%)	45.90±18.91	38.01±20.92	-2.87	188.62	**0.01**
**Macrolinguistic measures**					
Local coherence errors (ambiguous)	0.34±0.61	0.53±0.86	1.82	159.70	0.07
Local coherence errors (missings)	1.25±1.36	0.99±1.09	-.159	223.19	0.11
Utterances with semantic errors (words)	1.58±3.52	3.06±5.48	2.32	148.32	**0.02**

HC, Healthy Controls; FEP-NA, First Episode Psychosis–Non-Affective.

**Table 4:** Post-Hoc analysis (HC vs FEP-A).

	HC (N = 133)	FEP-A (N = 38)			
Microlinguistic measures	Mean ± st.dev.	Mean ± st.dev.	t-test	df	p-value
Speech-rate (words per minute)	134.29±25.31	119.11±32.56	-2.65	50.45	**0.01**
Mean length of utterances (words)	7.19 ±1.67	6.24±1.54	-3.23	63.57	**0.00**
Phonological paraphasias	0.05±0.26	0.08±0.36	0.42	48.23	0.68
Lexical fillers	7.02±8.97	5.00±6.88	-1.48	76.64	0.14
Syntactic completeness (%)	45.90±18.91	45.45±22.98	-0.07	51.89	0.94
**Macrolinguistic measures**					
Local coherence errors (ambiguous)	0.34±0.61	0.55±0.76	1.60	51.57	0.12
Local coherence errors (missings)	1.25±1.36	0.79±0.91	-2.43	89.81	**0.02**
Utterances with semantic errors (words)	1.58±3.52	4.05±14.20	1.06	38.30	0.29

HC, Healthy Controls; FEP-A, First Episode Psychosis—Affective.

**Table 5:** Post-Hoc analysis (FEP-A vs FEP-NA).

	FEP-A (N = 38)	FEP-NA (N = 95)			
Microlinguistic measures	Mean ± st.dev.	Mean ± st.dev.	t-test	df	p-value
Speech-rate (words per minute)	119.11±32.56	119.56±40.62	0.07	84.50	0.95
Mean length of utterances (words)	6.24±1.54	6.23±1.53	-0.02	67.56	0.98
Phonological paraphasias	0.08±0.36	0.16±0.42	1.09	79.42	0.28
Lexical fillers	5.00±6.88	4.63±7.91	-0.27	77.93	0.79
Syntactic completeness (%)	45.45±22.98	38.01±20.92	-1.73	62.88	0.09
**Macrolinguistic measures**					
Local coherence errors (ambiguous)	0.55±0.76	0.53±0.86	-0.17	76.74	0.86
Local coherence errors (missings)	0.79±0.91	0.99±1.09	1.09	81.32	0.28
Utterances with semantic errors (words)	4.05±14.20	3.06±5.48	-0.42	41.48	0.68

FEP-A, First Episode Psychosis–Affective; FEP-NA, First Episode Psychosis–Non-Affective.

### Results of neuropsychological assessment

All subgroups of patients with FEP had lower Verbal IQ (V-IQ) than HC (all p<0.05). Post-hoc comparisons showed that both FEP-NA and FEP-A had a lower V-IQ than HC (all p<0.02) and that FEP-NA and FEP-A did not differ between each other (p>0.02). Patients with FEP generally reported worse n-Back sensitivity scores than HC in all the difficulty conditions (0-Back, 1-Back, 2-Back, 3-Back, all p<0.05). The Post-hoc comparisons revealed a significant difference between FEP-NA and HC and FEP-A and HC in the 1-Back, 2-Back and 3-Back conditions (all p<0.02). Instead, the Post-hoc difference between FEP-NA and HC and FEP-A and HC did not pass the Bonferroni correction in the 0-Back condition (p>0.02). FEP-NA and FEP-A did not differ significantly between each other in any condition (all p>0.02). As for n-Back specificity, patients with FEP generally reported worse specificity scores than HC in the conditions 0-Back and 1-Back (p<0.05), while they did not differ in the 2-Back and 3-Back conditions (p>0.05). The Post-hoc comparisons revealed a significant difference between FEP-NA and HC in the 1-Back condition (p<0.02). All the other Post-hoc comparisons (i.e. FEP-A v HC; FEP-NA v FEP-A) were not significant (p>0.02). Concerning SOA sensitivity, FEPs’ patients did significantly worse than HC at both conditions, with 3 and 12 letters (all p<0.05). The Post-hoc comparisons between FEP-NAF and HC, as well as between FEP-NA and HC, highlighted significant differences (p<0.02). Instead, the comparison between FEP-NA and FEO-A did not prompt any significant difference (p>0.02). The analysis of SOA specificity scores showed a significant main difference between FEP and HC in the condition with 3 letters (p<0.05). The Post-hoc comparisons showed that FEP-NA had significantly poorer Specificity scores (3 letters condition) than HC (p<0.02). All the other comparisons did not give any significant result (all p>0.02). FEPs and HCs groups’ mean (+/- SD) neurocognitive scores are summarized in Table 6. Comparison between non-affective and affective FEP patients are detailed in S2 Table.

**Table 6:** Cognitive and neuropsychological data in FEP and HC.

	HC	FEP	Comparison
IQ (TIB)	120.25 ±5.50	110.83 ±7.73	t(195.8) = 10.74, p<0.00*
Span of Apprehension 3-trials, Sensitivity	0.48 ±0.02	0.42 ±0.12	t(146.0) = 5.06, p<0.00*
Span of Apprehension 12-trials, Sensitivity	0.35 ±0.09	0.27 ±0.12	t(178.9) = 5.39, p<0.00*
Span of Apprehension 3-trials, Specificity	0.94 ±0.05	0.87 ±0.22	t(151.9) = 3.50, p<0.00*
Span of Apprehension 12-trials, Specificity	0.87±0.13	0.84±0.20	t(188.8) = 1.37, p = 0.17
N-Back 0-trials, Sensitivity	0.99 ±0.04	0.96 ±0.12	t(122.6) = 2.61, p = 0.01*
N-Back 1-trials, Sensitivity	0.97 ±0.13	0.80 ±0.33	t(137.0) = 4.95, p<0.00*
N-Back 2-trials, Sensitivity	0.88 ±0.27	0.69 ±0.38	t(192.5) = 4.54, p<0.00*
N-Back 3-trials, Sensitivity	0.63 ±0.37	0.44 ±0.37	t(230.8) = 4.05, p<0.00*
N-Back 0-trials, Specificity	0.98 ±0.03	0.93 ±0.15	t(116.9) = 3.25, p<0.00*
N-Back 1-trials, Specificity	1.00 ±0.01	0.95 ±0.18	t(109.0) = 2.51, p = 0.01*
N-Back 2-trials, Specificity	1.00±0.02	0.97±0.16	t(111.5) = 1.86, p = 0.07
N-Back 3-trials, Specificity	0.98 ±0.05	0.95 ±0.14	t(125.7) = 1.92, p = 0.06

HC, Healthy Controls; FEP, First Episode Psychosis; IQ, Intelligence Quotient; TIB, Brief Intelligence Test.

### Correlation analysis results

We performed correlation analysis between the variables which resulted to be statistically significant in previous (Bonferroni corrected) t-tests on linguistic assessment. In the group of FEP, local coherence errors (both ambiguous and missing scores) negatively correlated with verbal IQ (p<0.05). In the Group of HC, local coherence errors (ambiguous) correlate negatively with SOA12 sensitivity (p<0.05). After correction (alpha level = 0.05 divided by the number of correlations), no correlations survived, neither in the group of FEP nor in the group of HC. Results are detailed as S3 Table.

### ML results

**Fig 1:**
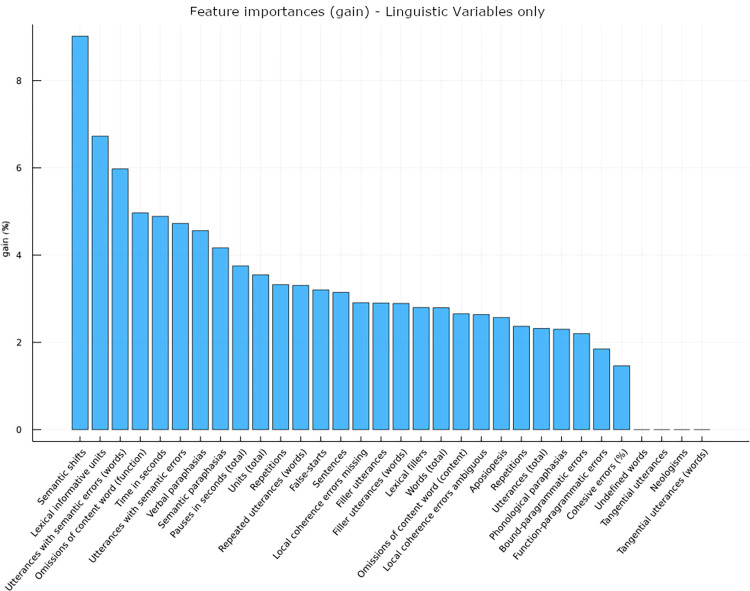
Language production variables feature gain.

Our model trained only on language production variables reached an accuracy of 76.36% in discriminating between FEP and HC. Fig 1 shows the relative importance of each linguistic variable in distinguishing FEP from HC in this model (XGBoost’s result feature gain). Specifically, the three variables with the highest predictive power are semantic shifts, followed by lexical informative units and utterances with semantic errors (Fig 1). Various metrics (precision, recall, f1-score and accuracy) predicting group condition obtained on several sets of variables including and/or combining linguistic data with sociodemographic (gender, age, educational level) and/or neuropsychological (SOA, n-Back, IQ verbal) measures are summarized in Table 7. ML analysis was not able to discriminate between FEP-NA and FEP-A, possibly due to the small and not balanced samples.

**Table 7:** XGBoost models precision, recall, f1-score and accuracy metrics by different predictive variables sets.

Features	precision	recall	f1-score	accuracy
**Sociodemographic set of measures**	79.87%	91.35%	85.04%	84.11%
**IQ TIB (verbal)**	78.57%	84.03%	80.83%	82.53%
**GAF**	97.97%	98.01%	97.94%	97.72%
**Linguistic production set of measures**	73.29%	63.75%	67.59%	70.04%
**SOA**	74.86%	93.40%	82.81%	75.05%
**nBack**	82.99%	64.15%	71.43%	77.27%
**Neuropsychological set of measures**	77.23%	92.78%	83.97%	75.51%
**IQ TIB (verbal) + Language production**	79.80%	82.45%	80.49%	82.39%
**SOA + Language production**	81.28%	86.19%	83.38%	78.18%
**nBack + Language production**	76.94%	67.65%	71.43%	75.69%

SOA, Span of Apprehension; GAF, Global Assessment of Functioning; IQ, Intelligence Quotient; TIB, Brief Intelligence Test

## Discussion

This study focused on the identification of potential language production difficulties in psychotic patients as early as in their first episode of disease. To the best of our knowledge, this is the first study assessing patients with FEP language production skills using a multilevel procedure for the analysis of narrative production abilities through a standardized procedure that aims at minimizing to the bone the subjective bias of scale raters thus reaching the highest reproducibility possible.

First, we evaluated whether patients with FEP would show deficits in linguistic performance compared to HC. Results showed that FEP patients have significant deficits in language production at the micro-level of speech, which includes speech measures making up for the intra-phrasal level of discourse construction. In particular, FEP patients had a productive style that was characterized by a “lower speech rate” and “shorter utterances” compared to HC, meaning that they verbalized fewer well-formed words per minute and constructed sentences which were composed of fewer words. Patients also showed a tendency to produce “more phonological paraphasias” than HC, meaning that the generally shorter sentences they produced also contain some errors, although the absolute values of paraphasias are very low in both HC and FEP to draw conclusive results. By contrast, impairments in speech rate and length of discourse are more relevant in absolute quantitative terms (119 vs 134 words per minute and 6 vs 7 word per sentence in FEP and HC, respectively). This is in line with other literature findings on psychotic patients, with poverty of speech being one of the most consistent findings in these patients, not only in chronic populations, but also at the onset of disease [5, 6, 18, 26]. Narratives of people with FEP also have a lower percentage of syntactic completeness, meaning that their discourses are not only shorter but also less complex with respect to controls. Moreover, our analysis shows a reduced use of lexical fillers by FEP compared to healthy subjects. Lexical fillers are meaningless words that we usually use during everyday colloquial speech and that interrupt the flow of a sentence by filling silent moments between ordinary (non-filler) words or sentences. For example, ‘kind of’ or ‘sort of’ or ‘y’know’ are common lexical fillers in the English language. Interestingly, fillers during speech occur when a person is engaged in verbal working memory and word retrieval tasks, being such mental operations anatomically sustained by large-scale brain networks encompassing associative cortices [56]. The fact that FEP uses fewer fillers than controls may suggest a difficulty in accessing verbal memory to retrieve well-formed words, which could at least partly explain the poverty of speech. Overall, our results on FEP show a similar pattern of micro-linguistic impairments to that observed in chronic patients with schizophrenia in a previous paper by our group using an analogue linguistic analysis [14]. Similarity refers not only to the types of linguistic abilities which are most impaired, but also to absolute values; in fact, the severity of the impairment does not change much between onset of disease and the chronic stages. Such similarity suggests that deficits at the micro-linguistic productive level would be constant through the course of illness, although more data should be collected on a direct comparison between the two populations to deepen this issue. In regards to macro-linguistic abilities, patients showed a slightly more ambiguous choice of those elements of a discourse which grant cohesion in sentences, i.e. by using words with unclear referents (‘he’ instead of ‘she’). The final effect for the listener is a reduction of the local coherence of the narration. Moreover, patients showed a tendency to construct utterances with more semantic errors than controls. However, while these differences were in fact statistically significant between FEP and HC, the absolute number of these types of error in both groups was little, especially when looking at the local coherence items. However, it is also meaningful that such impairments in the macro-linguistic level of linguistic processing are in line with previous literature findings in chronic [14] and FEP patients, both producing a more disorganized speech with aberrant use of connectives [30] and choosing more peculiar words than controls [26]. As for the second aim of our study, we subsequently performed post-hoc comparisons between FEP-A vs FEP-NA, FEP-NA vs HC and FEP-A vs HC. We considered language production to possibly be differently altered in affective and non-affective psychotic patients, given that the clinical presentation of these two types of patients is often significantly different and we already have some evidence from previous literature [21, 27, 57]. Interestingly, the analysis confirmed significant impairments in speech rate and mean length of utterances in both groups of affective and non-affective patients when compared to HC, strengthening the thesis that all psychotic patients show to some extent an impairment in same productive aspects of phrasal construction (speech rate and mean length of utterances), while other results were less consistent. When FEP-NA and FEP-A were put in direct comparisons, however, there was no significant difference between the two populations on language production. Nonetheless, while no difference was strong enough to be significant, patients in the FEP-NA group did display a relevantly lower percentage of syntactic completeness than those in the FEP-A group, which more or less performed as well as the HC group on this item. When put beside the results by Docherty et al [33], which found schizophrenic patients to produce utterances with a significantly lower syntactic complexity than patients with mania even at an early stage of illness, findings may point towards the hypothesis that patients in the schizophrenia-spectrum, even at onset of disease, show difficulties in the syntactic level of language production. Nevertheless, more data is needed to further evaluate this matter.

We also hypothesized that language production abilities of our patients would be to some extent independent from other cognitive impairments, while some others (e.g., language planning and discourse organization) would be more related to cognitive skills (e.g., executive functions). Patients with FEP did indeed perform worse than controls in our neuropsychological tasks (verbal-IQ, n-back, SOA), in line with current literature [37, 58–66]. However, in our sample, the language deficits observed in FEP and HC did not appear to be related to working memory and to the ability to process contextual information, after correction for multiple comparisons. This might point toward the idea that language production impairment at the onset of the illness appears to not be subdued to impairments in other cognitive abilities, but it is perhaps independent (at least the variables that we have explored and whose main effect in t-tests are statistically significant). More research should be conducted on this matter in order to confirm these results and reach a greater understanding of their causes.

Finally, the last aim of our study was to evaluate whether language production measures could be used to build a predictive model discriminating between FEP and HC. We therefore trained several ML models using different sets of features, also including clinical, socio-demographic and neuropsychological measures. Firstly, prediction accuracy of the model using language production variables only reaches 76.36%. This is quite interesting, when considering that the dummy classifier usually has a prediction accuracy of around 60%. Among the linguistic variables, those with the highest accuracy in predicting groups (FEP vs HC) were semantic shifts (it occurred when the concept in the interrupted preceding utterance was not resumed in the following sentence), lexical informative units (words that were not only well-formed from a phonological point of view, but also grammatically and pragmatically accurate) and utterances with semantic errors (Fig 1). Overall, such result points at a difficulty in the group of FEP in accessing semantically appropriate and accurately formed words and maintaining discourse coherence, which is in line with the results already described with reference to the first aim of the study (in particular, the reduced use of lexical fillers and the impairment in local coherence). Secondly, our ML results showed that GAF alone can predict the groups of FEP and HC with an accuracy of 97.90%. Also, neuropsychological measures have a predictive power of 99%. At a first glance, such results do not seem in favor of linguistic data, but some crucial issues should be considered: 1) although it is very easy and brief to administer, the Global Assessment of Functioning Scale score is (as the name suggests) a global measure, not specifically focused on psychosis but rather on the level of severity of symptoms and/or functioning characterizing several psychopathological conditions (ranging from personality disorders to anxiety, psychosis etc). The limited specificity of this scale and the impossibility to clearly distinguish between symptoms and functioning (although related) limit the informative power of this scale and its score; 2) the set of neuropsychological variables used in our ML analyses included SOA, n-back, and verbal-IQ. When combined together, their predictive power reached 99%, but such a result implies having a pc available (we used computerized versions of SOA and n-back), the presence of a neuropsychologist as part of the clinical staff and an assessment of around 1 hour. If, by contrast, we consider the single neuropsychological variable/instrument, the predictive power decreases to 76.67% for the SOA and 79.76% in the case of n-back, being these values comparable to the 76.36% reached by our set of linguistic data. Finally, Verbal IQ is 85.31% predictive with respect to the groups (FEP and HC) but, like GAF, it still represents a general measure. Based on such premises, we think that the use of language in the assessment of FEP and in classifying groups represents an extremely useful tool. Specifically, we think that linguistic deficits represent a core dimension of psychosis, being present both at the first stages and in the chronic phase of the illness and covering a large range of linguistic dimensions (both receptive and productive), as showed by a series of publications by our group [13–17] and elsewhere [i.e. 27]. In the present study, we used a very brief and simple task which consisted in the description by participants of a series of vignettes for a total of 10–30 seconds. Despite the simplicity and brevity of the task, the linguistic variables we used performed as well as the n-back in predicting groups. We also tried to build a predictive model that could discriminate between FEP-NA and FEP-A patients on all the same models performed to discriminate between FEP and HC, with no conclusive results. The attempt to predict the groups in this case did not indeed perform better than the dummy classifier, possibly due to the small number of subjects and the excessive imbalance in the size of the two groups (95 vs 38 subjects, respectively). Taken together, these results support the hypothesis that language production skills are impaired in patients with FEP at both micro- and macro-linguistic levels, being such deficits not related to other cognitive domains in our sample. Furthermore, semantic deficits were the most predictive of the group of FEP vs HC in the ML analyses. Importantly, the use of a narrative production task and of a multilevel procedure for the analysis of narrative discourse production allowed us to assess language in an ecological setting. This also allowed us to avoid the results’ biases highlighted by Barch and Berenbaum [67], with tasks with fewer directions yielding more negative thought disorders and tasks with vague topics yielding more positive thought disorders. Furthermore, studies such as the current investigation point toward the auspicial transition from a clinical practice based on clinical observation alone to the much more reliable “measured-based care” [68], with the adoption of a systematic analysis of language and which classifies patients on objective features using ML. On the other hand, our study also has some limitations. Firstly, because the patients were recruited within a larger project, they were all outpatients. This means that patients who had a psychotic episode at its worst were not evaluated in this study. Indeed, patients’ GAF scores were only moderately impaired. Our results should not then be considered definitive and representative of the whole population of patients with FEP. Also, the absence of significant differences between language production impairments in FEP-A and FEP-NA as shown by statistical analysis is not at all to be seen as conclusive, and further research should be done on the matter. As for ML analysis, the difference in sample sizes between FEP-A and FEP-NA (38 vs. 95) unfortunately did not allow us to obtain conclusive results. Balancing the datasets for FEP-A and FEP-NA was not possible because of the reduced sample size, as it would have produced unreliable results. As a limitation of our approach, it has also to be mentioned that we applied a time-consuming linguistic analysis method, which limits its application in clinical context. Despite that, we still support the importance of including such assessment in the first phases of psychosis. In particular, the recent advance of automatic methods for the analysis of natural samples of speech can tremendously reduce this limit (see [27] for an extensive review of such methods). Finally, our analysis can represent a first step for the construction of new linguistic tools based on the most predictive linguistic variables as shown by our statistical and ML analyses.

## Supporting information

S1 File Language production analysis. (DOCX)

S2 File Cognitive tasks. (DOCX)

S1 Table Sociodemographic and clinical data of the sample of FEP-A and FEP-NA. FEP-A, First Episode Psychosis–Affective; FEP-NA, First Episode Psychosis–Non-Affective; GAF, Global Assessment of Functioning; PANSS, Positive and Negative Syndrome Scale, General psychopathology subscale; HAM-D, Hamilton’s Depression Rating Scale; BRMRS, Bech-Rafaelsen Mania Rating Scale; DUP, Duration of untreated psychosis.
(DOCX)

S2 Table Cognitive and neuropsychological data in FEP-A and FEP-A. FEP-A, First Episode Psychosis–Affective; FEP-NA, First Episode Psychosis–Non-Affective; IQ, Intelligence Quotient; TIB, Brief Intelligence Test.
(DOCX)

S3 Table Correlations between linguistic and neuropsychological variables. (DOCX)

S4 Table Dataset. (DOCX)
