# Supplementary material for: Language production impairments in patients with a first episode of psychosis
Source: PLoS One. 2022 Aug 11;17(8):e0272873. doi: 10.1371/journal.pone.0272873 (PMC9371299; doi:10.1371/journal.pone.0272873)
Supplement: S1 File — (DOCX) [file pone.0272873.s001.docx]

**S2_Language production analysis**

Microlinguistic aspects of language analysis included an assessment of the participants’ productivity, lexical and grammatical processing. Here you can find a list of all the measures divided by areas of interest:

- Productivity
  - Speech Rate (i.e., number of words uttered in 1 minute);
  - Mean Length of Utterance (MLU; i.e., mean number of words per utterance);
- Lexical processing
  - % of phonological selection (i.e., percentage of phonologically well-formed words);
  - % of paragrammatic errors (i.e. percentage in the speech of erroneous bound-morphemes’ substitutions - e.g. those (plural) is a couple (singular) instead of this (singular) is a couple (singular), or function words (he is knocking from a door, instead of he is knocking on the door);
  - % of phonological and semantic paraphasias (i.e., percentage of errors consisting in substitutions of target words with other words that are phonologically or semantically similar);
  - Undefined words (i.e. percentage of unrecognizable words);
  - Neologisms (i.e., percentage of newly coined words referring to clearly identifiable objects or elements);
- Grammatical processing
  - % of omissions of content words (i.e., percentage of utterances containing lexical omissions, where one or more key elements of the scene are not clearly referred to);
  - Syntactic completeness (i.e., percentage of grammatically well-formed utterances)

The macrolinguistic assessment included measures of:

- Informative content
  - Lexical informativeness (i.e. percentage of words that were phonologically, grammatically and pragmatically accurate, which were not phonological errors, semantic paraphasias, paragrammatic errors, not ambiguous, repeated, or forming tangential utterances);
  - Utterances with semantic errors (i.e. percentage of utterances that failed in conveying the correct meaning because of any lack of lexical informativeness);
  - Repetitions of contents;
  - Repeated utterances;
- Textual organization
  - Cohesive errors (i.e., percentage of utterances that were not coherently linked between each other because of abrupt interruptions or incorrect use of cohesive function words);
  - Local coherence errors (i.e., percentage of utterances whose parts are poorly connected because of coherence errors, such as interruptions of misuse of conjunctions);
  - Aposiopesis (i.e., percentage of abrupt interruptions of the speech);
  - Global coherence errors (i.e., percentage of elements causing poor semantic coherence in the speech, including the production of tangential utterances).
- Additional features
  - Time in pauses (i.e., percentage of pauses longer that 10 seconds);
  - False-starts;
  - Lexical fillers;
  - Filler utterances (i.e., number of lexical elements or utterances that have no narrative meaning);
