# Supplementary material for: Language production impairments in patients with a first episode of psychosis
Source: PLoS One. 2022 Aug 11;17(8):e0272873. doi: 10.1371/journal.pone.0272873 (PMC9371299; doi:10.1371/journal.pone.0272873)
Supplement: S2 File — (DOCX) [file pone.0272873.s002.docx]

**S3 - Cognitive tasks**

- The Test di Intelligenza Breve (TIB) is the Italian version of the British National Adult Reading Test (NART; Nelson, Hazel, 1982. The National Adult Reading Test (NART): test manual. Windsor: NFE-Nelson). It comprises 54 word with both regular and irregular spellings (i.e. ‘recondito’ on Italian; ‘aisle’ in British English). Participants are asked to read aloud the list of words; spelling errors are listed and, together with age, gender and year of education, are used to estimate participant’s total, verbal and performance IQ.
- The N-Back task is a classic paradigm of working memory evaluation. In this task, a single letter is presented at each trial. The task has 4 memory load conditions of increasing difficulty, from 0 back to 3 back and consists of 5 blocks of trials, presented as follows: 0-Back; 1-Back; 2-Back; 3-Back; 0-Back, with the 0-Back condition presented two times, one at the beginning and one at the end. At each trial, the participants indicate whether a letter matched the one seen at the n precedent trial. For instance, in the 2-Back block, the participant has to press a button if the letter that was displayed in a trial was identical to the one presented two trials before. Note that, in the 0-Back condition, the target item (letter ‘X’) is specified beforehand at the beginning of the 0-Back blocks. Each condition has 14 trials including 3 matching digits and 11 distractors. Correct matches are counted as a “correct hit”. When the participant missed answering to a correct match, the event is counted as a “miss”. Answers to incorrect items are counted as a “false alarm”. The number of distractors correctly ignored are counted as “correct rejections”. The following measures are derived: Sensitivity (i.e., total “correct hit”/ [total “correct hit” + total “miss”]); Specificity (i.e. total “correct rejections”/ total number of distractors); Accuracy (i.e. [total “correct hit” + total “correct rejections”]/ total number of trials).
- The Span Of Apprehension task (SOA) is used to measure the ability to process multiple units of information by short-term memory. Sets of 3 (easy condition) or 12 (difficult condition) letters are presented at each trial. Participants are instructed to hit one between two keys if the letter ’T’ (key ‘1’) or the letter ‘F’ (key ‘2’) is present within the set. Participants have to inhibit any answer if neither ’T’ or ‘F’ was present. The letters ’T’ or ‘F’ are never simultaneously present within the same set. The whole task consists of one single block of 128 trials, 64 in the easy and 64 in the difficult condition, presented in a pseudo-random order. Similar to the N-Back task (see above), “correct hit”, “correct rejections” (i.e. answers correctly inhibited), “false alarm” and “miss” are counted. Reaction times for “correct hit” and “false alarm” are also collected. The following measures were derived: Sensitivity (i.e., “correct hit”/ total number of trials containing ’T’ or ’F’); Specificity (i.e., “correct rejections”/total number of trials NOT containing ’T’ or ’F’); Precision (i.e., “correct hit”/ “correct hit”+ “false alarm”); Accuracy (i.e., [“correct hit”+ ”correct rejections”]/total number of trials).
