# Supplementary material for: Language production impairments in patients with a first episode of psychosis
Source: PLoS One. 2022 Aug 11;17(8):e0272873. doi: 10.1371/journal.pone.0272873 (PMC9371299; doi:10.1371/journal.pone.0272873)
Supplement: S1 Table — FEP-A, First Episode Psychosis–Affective; FEP-NA, First Episode Psychosis–Non-Affective; GAF, Global Assessment of Functioning; PANSS, Positive and Negative Syndrome Scale, General psychopathology subscale; HAM-D, Hamilton’s Depression Rating Scale; BRMRS, Bech-Rafaelsen Mania Rating Scale; DUP, Duration of untreated psychosis. (DOCX) [file pone.0272873.s003.docx]

**S1 Table. Sociodemographic and clinical data of the sample of FEP-A and FEP-NA**

|  | **FEP-A** | **FEP-NA** | **Comparison** |
| --- | --- | --- | --- |
| Age in years | 29.12±9.33 | 28.85±8.93 | t(65.61)=2.27, p=0.88 |
| Education in years | 12.05±3.22 | 11.39±2.98 | t(63.71)=1.08, p=0.28 |
| GAF | 53.05±13.60 | 43.59±11.60 | t(59.89)=3.73, p<0.00* |
| PANSS | 31.76±9.11 | 36.23 ±8.54 | t(64.41)=2.57, p=0.01* |
| HAM-D | 14.18±9.73 | 15.65±6.90 | t(52.55)=0.84, p=0.41 |
| BRMRS | 2.66±3.22 | 2.56±3.47 | t(73.10)=0.16, p=0.88 |
| DUP | 203.94±420.43 | 332.12±619.69 | t(89.38)=1.28, p=0.20 |

FEP-A, First Episode Psychosis – Affective; FEP-NA, First Episode Psychosis – Non-Affective; GAF, Global Assessment of Functioning; PANSS, Positive and Negative Syndrome Scale, General psychopathology subscale; HAM-D, Hamilton's Depression Rating Scale; BRMRS, Bech-Rafaelsen Mania Rating Scale; DUP, Duration of untreated psychosis.
