# Supplementary material for: Language production impairments in patients with a first episode of psychosis
Source: PLoS One. 2022 Aug 11;17(8):e0272873. doi: 10.1371/journal.pone.0272873 (PMC9371299; doi:10.1371/journal.pone.0272873)
Supplement: S2 Table — FEP-A, First Episode Psychosis–Affective; FEP-NA, First Episode Psychosis–Non-Affective; IQ, Intelligence Quotient; TIB, Brief Intelligence Test. (DOCX) [file pone.0272873.s004.docx]

**S4 Table. Cognitive and neuropsychological data in FEP-A and FEP-A**

|  | **FEP-A** | **FEP-NA** | **Comparison** |
| --- | --- | --- | --- |
| IQ (TIB) | 112.41±7.37 | 110.11±7.79 | t(69.25)=1.49, p=0.14 |
| Span of Apprehension 3-trials, Sensitivity | 0.44±0.09 | 0.42±0.13 | t(84.54)=1.06, p=0.29 |
| Span of Apprehension 12-trials, Sensitivity | 0.28±0.10 | 0.26±0.13 | t(76.64)=1.12, p=0.27 |
| Span of Apprehension 3-trials, Specificity | 0.88±0.21 | 0.87±0.23 | t(84.54)=0.45, p=0.65 |
| N-Back 0-trials, Sensitivity | 0.95±0.14 | 0.97±0.11 | t(51.17)=0.89, p=0.38 |
| N-Back 1-trials, Sensitivity | 0.84±0.26 | 0.79±0.35 | t(84.81)=0.93, p=0.36 |
| N-Back 2-trials, Sensitivity | 0.61±0.36 | 0.72±0.38 | t(67.89)=1.47, p=0.15 |
| N-Back 3-trials, Sensitivity | 0.44±0.38 | 0.44±0.37 | t(63.28)=0.07, p=0.94 |
| N-Back 0-trials, Specificity | 0.96±0.04 | 0.92±0.17 | t(90.02)=1.57, p=0.12 |
| N-Back 1-trials, Specificity | 0.99±0.03 | 0.94±0.22 | t(79.84)=2.00, p=0.05 |

FEP-A, First Episode Psychosis – Affective; FEP-NA, First Episode Psychosis – Non-Affective; IQ, Intelligence Quotient; TIB, Brief Intelligence Test.
