# Supplementary material for: Language production impairments in patients with a first episode of psychosis
Source: PLoS One. 2022 Aug 11;17(8):e0272873. doi: 10.1371/journal.pone.0272873 (PMC9371299; doi:10.1371/journal.pone.0272873)
Supplement: S3 Table — (DOCX) [file pone.0272873.s005.docx]

**S5 Table. Correlations between linguistic and psychological variables**

**FEP**

| **variables** | **Pearsons'r** | **p-value** |
| --- | --- | --- |
| Speech Rate ~ 3-back sensitivity | 0,11959976 | 0,21545136 |
| Speech Rate ~ 3-back specificity | 0,008250335 | 0,932146575 |
| Speech Rate ~ SOA3 sensitivity | -0,045224026 | 0,60941081 |
| Speech Rate ~ SOA3 specificity | -0,053273634 | 0,54719139 |
| Speech Rate ~ SOA12 sensitivity | 0,03266388 | 0,712182099 |
| Speech Rate ~ SOA12 specificity | 0,025522515 | 0,773164672 |
| Speech Rate ~ QI TIB(verbal) | 0,09062785 | 0,341953413 |
| Mean length of utterances (words) ~ 3-back sensitivity | -0,04516414 | 0,640979758 |
| Mean length of utterances (words) ~ 3-back specificity | -0,046821372 | 0,62876978 |
| Mean length of utterances (words) ~ SOA3 sensitivity | 0,054308143 | 0,539426275 |
| Mean length of utterances (words) ~ SOA3 specificity | 0,123477888 | 0,161624323 |
| Mean length of utterances (words) ~ SOA12 sensitivity | -0,012955118 | 0,883691999 |
| Mean length of utterances (words) ~ SOA12 specificity | 0,062054724 | 0,483068651 |
| Mean length of utterances (words) ~ QI TIB(verbal) | 0,130390788 | 0,170592146 |
| Phonological paraphasias ~ 3-back sensitivity | 0,154136891 | 0,109540343 |
| Phonological paraphasias ~ 3-back specificity | 0,082730842 | 0,392415604 |
| Phonological paraphasias ~ SOA3 sensitivity | 0,085302017 | 0,334565176 |
| Phonological paraphasias ~ SOA3 specificity | 0,085880202 | 0,331287346 |
| Phonological paraphasias ~ SOA12 sensitivity | 0,037814565 | 0,669276587 |
| Phonological paraphasias ~ SOA12 specificity | 0,092734547 | 0,29399969 |
| Phonological paraphasias ~ QI TIB(verbal) | 0,044158583 | 0,643856577 |
| Lexical fillers ~ 3-back sensitivity | -0,008202001 | 0,932543155 |
| Lexical fillers ~ 3-back specificity | 0,095748691 | 0,321979316 |
| Lexical fillers ~ SOA3 sensitivity | 0,098825298 | 0,263293792 |
| Lexical fillers ~ SOA3 specificity | 0,041519164 | 0,639054241 |
| Lexical fillers ~ SOA12 sensitivity | 0,15877538 | 0,07118476 |
| Lexical fillers ~ SOA12 specificity | -0,113056205 | 0,200299191 |
| Lexical fillers ~ QI TIB(verbal) | 0,037116669 | 0,69762152 |
| Syntactic completeness (%) ~ 3-back sensitivity | 0,086106513 | 0,373321377 |
| Syntactic completeness (%) ~ 3-back specificity | -0,091432615 | 0,344372163 |
| Syntactic completeness (%) ~ SOA3 sensitivity | -0,003493677 | 0,968532067 |
| Syntactic completeness (%) ~ SOA3 specificity | 0,067100265 | 0,448135512 |
| Syntactic completeness (%) ~ SOA12 sensitivity | -0,014610479 | 0,868955805 |
| Syntactic completeness (%) ~ SOA12 specificity | 0,188410229 | 0,031815247 |
| Syntactic completeness (%) ~ QI TIB(verbal) | 0,134540596 | 0,157271033 |
| Local coherence errors (ambiguous) ~ 3-back sensitivity | 0,016271976 | 0,866632876 |
| Local coherence errors (ambiguous) ~ 3-back specificity | 0,008957437 | 0,926346809 |
| Local coherence errors (ambiguous) ~ SOA3 sensitivity | 0,022760747 | 0,797148418 |
| Local coherence errors (ambiguous) ~ SOA3 specificity | 0,113734352 | 0,197595704 |
| Local coherence errors (ambiguous) ~ SOA12 sensitivity | 0,050357856 | 0,569367229 |
| Local coherence errors (ambiguous) ~ SOA12 specificity | -0,012735047 | 0,885654204 |
| Local coherence errors (ambiguous) ~ QI TIB(verbal) | -0,212452637 | 0,024518709 |
| Local coherence errors (missings) ~ 3-back sensitivity | 0,124552876 | 0,19690844 |
| Local coherence errors (missings) ~ 3-back specificity | 0,07413448 | 0,443605752 |
| Local coherence errors (missings) ~ SOA3 sensitivity | 0,051858262 | 0,557903071 |
| Local coherence errors (missings) ~ SOA3 specificity | 0,123577519 | 0,161283622 |
| Local coherence errors (missings) ~ SOA12 sensitivity | 0,059881515 | 0,498553278 |
| Local coherence errors (missings) ~ SOA12 specificity | 0,121815619 | 0,167388183 |
| Local coherence errors (missings) ~ QI TIB(verbal) | -0,306453122 | 0,001015294 |
| Utterances with semantic errors (words) ~ 3-back sensitivity | 0,027512381 | 0,776425764 |
| Utterances with semantic errors (words) ~ 3-back specificity | 0,040521313 | 0,675691614 |
| Utterances with semantic errors (words) ~ SOA3 sensitivity | 0,054761991 | 0,536036947 |
| Utterances with semantic errors (words) ~ SOA3 specificity | 0,089955395 | 0,308768937 |
| Utterances with semantic errors (words) ~ SOA12 sensitivity | 0,077504459 | 0,380773861 |
| Utterances with semantic errors (words) ~ SOA12 specificity | -0,05748416 | 0,515932811 |
| Utterances with semantic errors (words) ~ QI TIB(verbal) | 0,133586739 | 0,160261906 |

**HC**

| **variables** | **Pearsons'r** | **p-value** |
| --- | --- | --- |
| Speech Rate ~ 3-back sensitivity | 0,031547576 | 0,718488309 |
| Speech Rate ~ 3-back specificity | 0,053222488 | 0,542902776 |
| Speech Rate ~ SOA3 sensitivity | 0,061486613 | 0,613102348 |
| Speech Rate ~ SOA3 specificity | 0,112590433 | 0,353411187 |
| Speech Rate ~ SOA12 sensitivity | 0,057235387 | 0,637905399 |
| Speech Rate ~ SOA12 specificity | 0,188101249 | 0,118904924 |
| Speech Rate ~ QI TIB(verbal) | -0,032621067 | 0,70933259 |
| Mean length of utterances (words) ~ 3-back sensitivity | 0,096576812 | 0,268790594 |
| Mean length of utterances (words) ~ 3-back specificity | -0,04111276 | 0,638455994 |
| Mean length of utterances (words) ~ SOA3 sensitivity | -0,076598787 | 0,528525075 |
| Mean length of utterances (words) ~ SOA3 specificity | -0,087444509 | 0,471635445 |
| Mean length of utterances (words) ~ SOA12 sensitivity | 0,002130813 | 0,986032406 |
| Mean length of utterances (words) ~ SOA12 specificity | -0,022308967 | 0,854553043 |
| Mean length of utterances (words) ~ QI TIB(verbal) | -0,040032572 | 0,647309072 |
| Phonological paraphasias ~ 3-back sensitivity | -0,034580939 | 0,692724378 |
| Phonological paraphasias ~ 3-back specificity | 0,107864518 | 0,216525011 |
| Phonological paraphasias ~ SOA3 sensitivity | -0,087860515 | 0,469521168 |
| Phonological paraphasias ~ SOA3 specificity | -0,052020533 | 0,668878225 |
| Phonological paraphasias ~ SOA12 sensitivity | -0,005445037 | 0,964317348 |
| Phonological paraphasias ~ SOA12 specificity | 0,029680123 | 0,807304991 |
| Phonological paraphasias ~ QI TIB(verbal) | -0,101021377 | 0,247272083 |
| Lexical fillers ~ 3-back sensitivity | 0,006139992 | 0,944080557 |
| Lexical fillers ~ 3-back specificity | 0,025916429 | 0,76714507 |
| Lexical fillers ~ SOA3 sensitivity | 0,061186836 | 0,614837655 |
| Lexical fillers ~ SOA3 specificity | 0,045459789 | 0,708635845 |
| Lexical fillers ~ SOA12 sensitivity | 0,161515391 | 0,181617144 |
| Lexical fillers ~ SOA12 specificity | 0,110182668 | 0,363865254 |
| Lexical fillers ~ QI TIB(verbal) | -0,082777995 | 0,343507272 |
| Syntactic completeness (%) ~ 3-back sensitivity | 0,091364259 | 0,295603776 |
| Syntactic completeness (%) ~ 3-back specificity | -0,017526248 | 0,841300693 |
| Syntactic completeness (%) ~ SOA3 sensitivity | 0,006889389 | 0,954861001 |
| Syntactic completeness (%) ~ SOA3 specificity | 0,061680673 | 0,611980133 |
| Syntactic completeness (%) ~ SOA12 sensitivity | 0,067603813 | 0,578165329 |
| Syntactic completeness (%) ~ SOA12 specificity | -0,029447731 | 0,808785081 |
| Syntactic completeness (%) ~ QI TIB(verbal) | 0,049350137 | 0,572682565 |
| Local coherence errors (ambiguous) ~ 3-back sensitivity | -0,050360741 | 0,564837187 |
| Local coherence errors (ambiguous) ~ 3-back specificity | -0,003850116 | 0,964918166 |
| Local coherence errors (ambiguous) ~ SOA3 sensitivity | -0,138388884 | 0,253244537 |
| Local coherence errors (ambiguous) ~ SOA3 specificity | 0,012243252 | 0,919872973 |
| Local coherence errors (ambiguous) ~ SOA12 sensitivity | -0,293878468 | 0,013538453 |
| Local coherence errors (ambiguous) ~ SOA12 specificity | 0,137378394 | 0,256760413 |
| Local coherence errors (ambiguous) ~ QI TIB(verbal) | 0,038061321 | 0,663592286 |
| Local coherence errors (missings) ~ 3-back sensitivity | 0,025787681 | 0,768269058 |
| Local coherence errors (missings) ~ 3-back specificity | 0,150457089 | 0,083875075 |
| Local coherence errors (missings) ~ SOA3 sensitivity | 0,162481496 | 0,178983937 |
| Local coherence errors (missings) ~ SOA3 specificity | -0,068270331 | 0,574414659 |
| Local coherence errors (missings) ~ SOA12 sensitivity | 0,066006611 | 0,587198723 |
| Local coherence errors (missings) ~ SOA12 specificity | -0,06728764 | 0,579948444 |
| Local coherence errors (missings) ~ QI TIB(verbal) | -0,066400508 | 0,447626334 |
| Utterances with semantic errors (words) ~ 3-back sensitivity | 0,018205574 | 0,835234984 |
| Utterances with semantic errors (words) ~ 3-back specificity | -0,007395261 | 0,932672883 |
| Utterances with semantic errors (words) ~ SOA3 sensitivity | 0,0938423 | 0,439693179 |
| Utterances with semantic errors (words) ~ SOA3 specificity | 0,009313959 | 0,939001667 |
| Utterances with semantic errors (words) ~ SOA12 sensitivity | -0,200508684 | 0,09604873 |
| Utterances with semantic errors (words) ~ SOA12 specificity | 0,175924417 | 0,14517837 |
| Utterances with semantic errors (words) ~ QI TIB(verbal) | -0,079573383 | 0,362576054 |
