# Supplementary material for: Language production impairments in patients with a first episode of psychosis
Source: PLoS One. 2022 Aug 11;17(8):e0272873. doi: 10.1371/journal.pone.0272873 (PMC9371299; doi:10.1371/journal.pone.0272873)
Supplement: S4 Table — (DOCX) [file pone.0272873.s006.docx]

**S6 Table. Dataset**

| Group (FEP=0;CNT=1) | Age | Sex | Narrative time | Pauses (seconds) | Units | Clauses | Words | Phonological paraphasias | Semantic paraphasias | Verbal paraphasias | Lexical fillers | Fillers (clauses) | Fillers (words) |
| --- | --- | --- | --- | --- | --- | --- | --- | --- | --- | --- | --- | --- | --- |
| 1 | 30 | M | 50 | 4 | 127 | 23 | 124 | 0 | 1 | 0 | 3 | 2 | 8 |
| 0 | 19 | M | 15 | 0 | 33 | 5 | 33 | 0 | 0 | 0 | 0 | 0 | 0 |
| 0 | 21 | M | 15 | 0 | 41 | 7 | 39 | 0 | 0 | 0 | 0 | 0 | 0 |
| 0 | 34 | F | 53 | 10 | 95 | 15 | 94 | 0 | 0 | 0 | 6 | 1 | 16 |
| 1 | 29 | F | 100 | 4 | 164 | 25 | 163 | 0 | 0 | 0 | 15 | 2 | 11 |
| 1 | 22 | F | 43 | 6 | 116 | 21 | 115 | 0 | 0 | 0 | 2 | 2 | 10 |
| 1 | 40 | M | 32 | 0 | 86 | 8 | 86 | 0 | 0 | 0 | 8 | 0 | 0 |
| 1 | 28 | M | 73 | 17 | 167 | 31 | 165 | 0 | 0 | 0 | 3 | 0 | 0 |
| 1 | 30 | F | 44 | 3 | 108 | 15 | 107 | 0 | 0 | 0 | 0 | 2 | 14 |
| 1 | 22 | F | 37 | 2 | 92 | 17 | 91 | 0 | 0 | 0 | 6 | 1 | 1 |
| 0 | 27 | M | 21 | 3 | 35 | 7 | 35 | 0 | 0 | 0 | 0 | 0 | 0 |
| 0 | 20 | F | 63 | 15 | 90 | 17 | 88 | 0 | 0 | 0 | 0 | 1 | 7 |
| 0 | 44 | F | 55 | 2 | 97 | 11 | 94 | 0 | 0 | 0 | 1 | 1 | 2 |
| 0 | 25 | M | 69 | 12 | 100 | 29 | 96 | 0 | 1 | 1 | 25 | 8 | 45 |
| 0 | 30 | M | 60 | 5 | 104 | 26 | 100 | 0 | 1 | 1 | 4 | 1 | 1 |
| 0 | 39 | M | 129 | 41 | 133 | 20 | 132 | 0 | 3 | 0 | 2 | 3 | 22 |
| 1 | 47 | F | 55 | 0 | 122 | 16 | 122 | 0 | 1 | 0 | 2 | 3 | 11 |
| 0 | 18 | M | 63 | 19 | 94 | 14 | 94 | 0 | 0 | 0 | 0 | 0 | 0 |
| 1 | 30 | F | 40 | 13 | 95 | 18 | 95 | 0 | 0 | 0 | 0 | 3 | 11 |
| 0 | 20 | F | 90 | 7 | 145 | 18 | 145 | 0 | 0 | 0 | 8 | 0 | 0 |
| 1 | 28 | F | 33 | 0 | 76 | 10 | 74 | 1 | 0 | 0 | 3 | 0 | 0 |
| 1 | 22 | M | 69 | 2 | 160 | 18 | 158 | 0 | 4 | 0 | 10 | 1 | 7 |
| 0 | 27 | M | 26 | 2 | 31 | 6 | 31 | 0 | 1 | 0 | 0 | 1 | 6 |
| 0 | 42 | F | 45 | 10 | 71 | 14 | 71 | 0 | 1 | 0 | 0 | 1 | 4 |
| 1 | 42 | M | 63 | 15 | 97 | 14 | 97 | 0 | 0 | 0 | 9 | 2 | 12 |
| 0 | 30 | M | 35 | 8 | 50 | 11 | 48 | 0 | 1 | 0 | 0 | 0 | 0 |
| 0 | 24 | F | 34 | 2 | 74 | 11 | 72 | 1 | 0 | 0 | 0 | 0 | 0 |
| 0 | 33 | F | 71 | 0 | 159 | 24 | 159 | 0 | 0 | 0 | 3 | 3 | 14 |
| 1 | 27 | M | 88 | 8 | 235 | 32 | 234 | 0 | 0 | 0 | 0 | 4 | 47 |
| 0 | 18 | F | 36 | 0 | 95 | 10 | 95 | 0 | 0 | 0 | 31 | 0 | 0 |
| 1 | 27 | F | 49 | 0 | 102 | 11 | 100 | 0 | 0 | 0 | 8 | 0 | 0 |
| 0 | 27 | M | 77 | 9 | 161 | 30 | 155 | 0 | 0 | 0 | 5 | 3 | 15 |
| 1 | 39 | F | 78 | 2 | 168 | 15 | 167 | 0 | 0 | 0 | 21 | 1 | 6 |
| 1 | 43 | M | 25 | 0 | 62 | 10 | 61 | 0 | 0 | 0 | 1 | 0 | 0 |
| 1 | 31 | M | 55 | 0 | 118 | 16 | 117 | 0 | 0 | 0 | 14 | 0 | 0 |
| 1 | 49 | F | 39 | 6 | 95 | 10 | 95 | 0 | 0 | 0 | 0 | 5 | 36 |
| 1 | 26 | F | 63 | 3 | 151 | 19 | 150 | 0 | 0 | 0 | 17 | 3 | 14 |
| 0 | 26 | F | 45 | 15 | 71 | 11 | 70 | 0 | 0 | 0 | 5 | 2 | 6 |
| 0 | 27 | M | 47 | 13 | 75 | 16 | 75 | 0 | 0 | 0 | 11 | 1 | 2 |
| 0 | 30 | M | 51 | 0 | 114 | 14 | 114 | 0 | 1 | 1 | 5 | 0 | 0 |
| 1 | 54 | F | 54 | 5 | 83 | 12 | 82 | 0 | 0 | 0 | 1 | 1 | 3 |
| 1 | 35 | F | 40 | 7 | 81 | 9 | 80 | 0 | 0 | 0 | 5 | 0 | 0 |
| 1 | 21 | F | 32 | 3 | 64 | 10 | 64 | 0 | 0 | 0 | 6 | 1 | 2 |
| 1 | 30 | F | 38 | 8 | 87 | 11 | 87 | 0 | 0 | 0 | 0 | 0 | 0 |
| 0 | 30 | F | 51 | 15 | 102 | 16 | 100 | 0 | 0 | 1 | 11 | 1 | 1 |
| 1 | 23 | F | 34 | 2 | 72 | 14 | 71 | 0 | 0 | 1 | 4 | 1 | 5 |
| 0 | 19 | F | 65 | 11 | 100 | 23 | 96 | 0 | 0 | 0 | 6 | 0 | 0 |
| 1 | 53 | F | 67 | 4 | 118 | 19 | 116 | 0 | 0 | 0 | 2 | 3 | 11 |
| 1 | 22 | F | 65 | 6 | 159 | 23 | 158 | 0 | 0 | 0 | 1 | 1 | 18 |
| 1 | 23 | F | 60 | 4 | 151 | 24 | 149 | 0 | 0 | 0 | 20 | 3 | 8 |
| 0 | 19 | M | 33 | 4 | 49 | 7 | 49 | 0 | 1 | 0 | 1 | 1 | 2 |
| 1 | 30 | M | 16 | 0 | 42 | 8 | 41 | 1 | 0 | 0 | 0 | 0 | 0 |
| 1 | 24 | F | 47 | 13 | 87 | 20 | 87 | 0 | 0 | 0 | 1 | 0 | 0 |
| 0 | 19 | M | 40 | 0 | 79 | 16 | 76 | 0 | 0 | 0 | 0 | 1 | 6 |
| 0 | 38 | F | 91 | 2 | 190 | 30 | 187 | 0 | 0 | 1 | 14 | 3 | 9 |
| 1 | 56 | M | 50 | 10 | 92 | 10 | 92 | 0 | 0 | 0 | 15 | 0 | 0 |
| 0 | 20 | F | 41 | 25 | 62 | 11 | 62 | 0 | 0 | 0 | 0 | 1 | 6 |
| 0 | 24 | F | 56 | 2 | 104 | 19 | 99 | 0 | 0 | 0 | 11 | 1 | 6 |
| 0 | 30 | M | 40 | 6 | 114 | 17 | 114 | 0 | 0 | 0 | 15 | 5 | 25 |
| 0 | 40 | F | 40 | 15 | 62 | 9 | 62 | 0 | 0 | 0 | 0 | 1 | 5 |
| 1 | 31 | M | 26 | 0 | 85 | 10 | 84 | 0 | 2 | 0 | 8 | 1 | 8 |
| 0 | 26 | M | 18 | 0 | 60 | 8 | 60 | 0 | 0 | 0 | 0 | 0 | 0 |
| 1 | 45 | M | 117 | 22 | 245 | 33 | 239 | 0 | 0 | 0 | 19 | 10 | 67 |
| 1 | 30 | M | 40 | 3 | 81 | 13 | 81 | 0 | 1 | 0 | 3 | 1 | 6 |
| 1 | 25 | F | 74 | 12 | 183 | 30 | 182 | 0 | 0 | 0 | 16 | 7 | 29 |
| 0 | 20 | M | 20 | 0 | 36 | 6 | 36 | 0 | 0 | 0 | 3 | 0 | 0 |
| 0 | 27 | M | 49 | 19 | 71 | 15 | 71 | 0 | 0 | 0 | 1 | 0 | 0 |
| 1 | 28 | M | 94 | 12 | 190 | 22 | 187 | 0 | 2 | 0 | 14 | 4 | 32 |
| 0 | 28 | M | 29 | 4 | 56 | 11 | 54 | 1 | 0 | 0 | 0 | 1 | 5 |
| 1 | 32 | M | 68 | 15 | 115 | 16 | 114 | 0 | 0 | 1 | 1 | 0 | 0 |
| 0 | 29 | F | 131 | 53 | 203 | 32 | 203 | 0 | 1 | 0 | 7 | 4 | 23 |
| 1 | 49 | F | 16 | 0 | 47 | 8 | 47 | 0 | 0 | 0 | 0 | 0 | 0 |
| 0 | 18 | M | 11 | 0 | 26 | 5 | 26 | 0 | 0 | 0 | 0 | 0 | 0 |
| 1 | 31 | M | 41 | 4 | 130 | 22 | 129 | 0 | 2 | 0 | 4 | 3 | 19 |
| 0 | 19 | M | 18 | 0 | 55 | 8 | 55 | 0 | 0 | 0 | 0 | 0 | 0 |
| 0 | 21 | M | 51 | 32 | 68 | 11 | 68 | 0 | 2 | 0 | 4 | 0 | 0 |
| 1 | 61 | M | 93 | 5 | 168 | 19 | 166 | 0 | 0 | 0 | 12 | 2 | 25 |
| 0 | 18 | M | 29 | 0 | 73 | 7 | 73 | 0 | 0 | 0 | 20 | 0 | 0 |
| 0 | 35 | M | 19 | 5 | 47 | 8 | 45 | 1 | 0 | 0 | 0 | 0 | 0 |
| 1 | 27 | M | 26 | 2 | 52 | 8 | 52 | 0 | 0 | 0 | 0 | 0 | 0 |
| 1 | 37 | F | 44 | 0 | 109 | 15 | 108 | 0 | 0 | 0 | 18 | 2 | 5 |
| 1 | 39 | M | 44 | 2 | 123 | 19 | 121 | 0 | 0 | 1 | 2 | 0 | 0 |
| 1 | 28 | F | 50 | 13 | 91 | 12 | 90 | 0 | 0 | 0 | 1 | 0 | 0 |
| 0 | 45 | M | 16 | 0 | 39 | 6 | 39 | 0 | 0 | 0 | 1 | 0 | 0 |
| 1 | 29 | F | 81 | 4 | 204 | 26 | 202 | 0 | 0 | 0 | 35 | 4 | 17 |
| 1 | 42 | F | 31 | 0 | 87 | 13 | 67 | 0 | 0 | 0 | 1 | 0 | 0 |
| 1 | 25 | F | 81 | 12 | 210 | 28 | 205 | 1 | 0 | 0 | 27 | 3 | 30 |
| 0 | 20 | M | 25 | 23 | 169 | 28 | 164 | 0 | 2 | 1 | 1 | 1 | 8 |
| 0 | 20 | F | 26 | 8 | 48 | 11 | 48 | 0 | 0 | 0 | 1 | 0 | 0 |
| 0 | 19 | M | 51 | 22 | 80 | 10 | 80 | 0 | 0 | 0 | 0 | 0 | 0 |
| 0 | 30 | M | 95 | 38 | 113 | 15 | 113 | 0 | 0 | 0 | 12 | 0 | 0 |
| 0 | 23 | M | 33 | 6 | 66 | 8 | 66 | 0 | 0 | 0 | 1 | 0 | 0 |
| 0 | 24 | M | 31 | 0 | 75 | 12 | 75 | 0 | 1 | 0 | 1 | 0 | 0 |
| 0 | 28 | M | 47 | 7 | 84 | 13 | 83 | 1 | 1 | 1 | 1 | 0 | 0 |
| 1 | 37 | M | 64 | 19 | 94 | 11 | 94 | 0 | 0 | 0 | 6 | 1 | 22 |
| 1 | 22 | M | 24 | 0 | 70 | 14 | 68 | 0 | 0 | 0 | 0 | 0 | 0 |
| 1 | 43 | M | 54 | 6 | 95 | 19 | 94 | 0 | 0 | 0 | 6 | 3 | 21 |
| 1 | 30 | F | 48 | 10 | 94 | 16 | 94 | 0 | 0 | 0 | 2 | 2 | 14 |
| 1 | 35 | M | 25 | 2 | 74 | 7 | 73 | 0 | 0 | 0 | 1 | 0 | 0 |
| 1 | 36 | M | 55 | 16 | 85 | 15 | 85 | 0 | 0 | 0 | 5 | 1 | 4 |
| 0 | 19 | M | 40 | 8 | 62 | 10 | 62 | 0 | 0 | 0 | 1 | 1 | 3 |
| 0 | 16 | F | 45 | 5 | 76 | 13 | 75 | 0 | 0 | 0 | 1 | 1 | 1 |
| 1 | 43 | F | 73 | 9 | 117 | 20 | 114 | 0 | 0 | 0 | 6 | 0 | 0 |
| 1 | 56 | M | 38 | 2 | 82 | 14 | 82 | 0 | 0 | 0 | 2 | 0 | 0 |
| 1 | 25 | M | 81 | 2 | 199 | 22 | 199 | 0 | 2 | 0 | 15 | 1 | 2 |
| 1 | 47 | M | 40 | 0 | 88 | 18 | 86 | 0 | 0 | 0 | 10 | 1 | 4 |
| 0 | 40 | F | 68 | 7 | 114 | 15 | 113 | 1 | 0 | 0 | 0 | 0 | 0 |
| 0 | 23 | M | 52 | 13 | 91 | 14 | 90 | 0 | 1 | 0 | 6 | 0 | 0 |
| 0 | 42 | F | 54 | 0 | 106 | 16 | 106 | 0 | 0 | 0 | 5 | 1 | 4 |
| 1 | 41 | F | 25 | 2 | 82 | 12 | 81 | 0 | 0 | 0 | 3 | 0 | 0 |
| 1 | 32 | F | 66 | 10 | 121 | 20 | 121 | 0 | 2 | 0 | 1 | 1 | 14 |
| 1 | 26 | M | 60 | 12 | 90 | 9 | 90 | 0 | 0 | 0 | 15 | 0 | 0 |
| 0 | 35 | M | 37 | 0 | 58 | 10 | 58 | 0 | 0 | 0 | 0 | 0 | 0 |
| 0 | 24 | F | 26 | 0 | 73 | 9 | 73 | 0 | 0 | 1 | 0 | 0 | 0 |
| 1 | 25 | F | 71 | 2 | 155 | 20 | 152 | 0 | 1 | 0 | 7 | 2 | 2 |
| 1 | 22 | M | 32 | 0 | 92 | 10 | 91 | 0 | 0 | 0 | 1 | 0 | 0 |
| 0 | 47 | F | 24 | 2 | 63 | 10 | 63 | 0 | 1 | 0 | 4 | 0 | 0 |
| 1 | 32 | F | 44 | 0 | 129 | 17 | 129 | 0 | 0 | 0 | 9 | 3 | 24 |
| 0 | 20 | M | 20 | 2 | 45 | 8 | 44 | 0 | 0 | 1 | 3 | 0 | 0 |
| 0 | 26 | M | 72 | 7 | 193 | 24 | 189 | 2 | 1 | 0 | 28 | 4 | 15 |
| 0 | 37 | F | 29 | 4 | 81 | 15 | 81 | 0 | 0 | 0 | 2 | 0 | 0 |
| 0 | 30 | M | 126 | 4 | 234 | 26 | 226 | 1 | 0 | 2 | 48 | 0 | 0 |
| 1 | 26 | M | 74 | 13 | 135 | 13 | 135 | 0 | 0 | 1 | 3 | 0 | 0 |
| 0 | 19 | M | 43 | 20 | 65 | 13 | 65 | 0 | 0 | 0 | 1 | 0 | 0 |
| 0 | 25 | F | 55 | 0 | 114 | 16 | 114 | 0 | 0 | 0 | 6 | 3 | 9 |
| 1 | 60 | M | 21 | 2 | 44 | 7 | 44 | 0 | 0 | 0 | 5 | 0 | 0 |
| 0 | 21 | M | 47 | 11 | 75 | 7 | 75 | 0 | 3 | 0 | 8 | 1 | 6 |
| 0 | 42 | M | 21 | 0 | 31 | 4 | 31 | 0 | 1 | 0 | 0 | 0 | 0 |
| 0 | 30 | M | 22 | 3 | 51 | 6 | 51 | 0 | 0 | 0 | 0 | 0 | 0 |
| 0 | 40 | M | 22 | 2 | 50 | 8 | 50 | 0 | 0 | 0 | 0 | 0 | 0 |
| 1 | 29 | F | 70 | 2 | 150 | 17 | 149 | 0 | 0 | 0 | 0 | 3 | 25 |
| 1 | 45 | F | 53 | 25 | 88 | 17 | 87 | 0 | 0 | 0 | 1 | 0 | 0 |
| 1 | 40 | F | 52 | 9 | 122 | 18 | 121 | 0 | 0 | 0 | 3 | 1 | 11 |
| 0 | 26 | F | 22 | 6 | 60 | 10 | 60 | 0 | 0 | 1 | 0 | 1 | 11 |
| 1 | 26 | F | 60 | 11 | 147 | 24 | 144 | 0 | 0 | 0 | 1 | 0 | 0 |
| 1 | 27 | F | 28 | 0 | 68 | 12 | 65 | 0 | 0 | 0 | 1 | 0 | 0 |
| 0 | 21 | M | 22 | 6 | 38 | 9 | 38 | 0 | 0 | 0 | 0 | 0 | 0 |
| 1 | 33 | M | 70 | 10 | 174 | 19 | 173 | 0 | 0 | 0 | 34 | 1 | 10 |
| 1 | 26 | F | 42 | 0 | 88 | 13 | 87 | 0 | 1 | 0 | 0 | 1 | 1 |
| 1 | 27 | M | 31 | 3 | 100 | 10 | 100 | 0 | 0 | 0 | 13 | 1 | 7 |
| 0 | 24 | M | 62 | 23 | 80 | 13 | 78 | 0 | 0 | 0 | 0 | 0 | 0 |
| 1 | 28 | M | 65 | 8 | 164 | 24 | 164 | 0 | 1 | 0 | 33 | 6 | 32 |
| 0 | 27 | M | 27 | 0 | 48 | 10 | 48 | 0 | 3 | 0 | 0 | 1 | 3 |
| 1 | 30 | F | 48 | 0 | 80 | 15 | 79 | 0 | 0 | 0 | 0 | 0 | 0 |
| 0 | 29 | M | 168 | 23 | 327 | 31 | 326 | 1 | 0 | 0 | 20 | 4 | 51 |
| 0 | 23 | F | 32 | 0 | 73 | 11 | 73 | 0 | 0 | 0 | 1 | 1 | 8 |
| 1 | 29 | F | 69 | 9 | 134 | 26 | 134 | 0 | 0 | 0 | 9 | 6 | 27 |
| 0 | 21 | F | 52 | 0 | 140 | 26 | 138 | 0 | 0 | 0 | 4 | 0 | 0 |
| 1 | 26 | F | 69 | 0 | 140 | 16 | 138 | 0 | 0 | 0 | 24 | 0 | 0 |
| 1 | 64 | M | 16 | 0 | 46 | 8 | 46 | 0 | 0 | 0 | 0 | 0 | 0 |
| 0 | 42 | M | 27 | 0 | 56 | 9 | 55 | 0 | 0 | 0 | 3 | 0 | 0 |
| 0 | 18 | M | 16 | 2 | 38 | 5 | 38 | 0 | 0 | 0 | 0 | 0 | 0 |
| 1 | 25 | F | 50 | 0 | 107 | 14 | 106 | 0 | 0 | 0 | 9 | 0 | 0 |
| 1 | 42 | M | 21 | 5 | 33 | 9 | 31 | 0 | 0 | 0 | 0 | 0 | 0 |
| 0 | 23 | M | 19 | 0 | 48 | 10 | 48 | 0 | 0 | 0 | 0 | 0 | 0 |
| 1 | 24 | F | 42 | 0 | 126 | 18 | 125 | 0 | 0 | 0 | 4 | 1 | 7 |
| 0 | 43 | M | 27 | 4 | 43 | 7 | 43 | 0 | 0 | 0 | 1 | 2 | 12 |
| 1 | 25 | M | 22 | 2 | 49 | 7 | 48 | 0 | 0 | 0 | 3 | 0 | 0 |
| 0 | 19 | F | 61 | 2 | 149 | 23 | 147 | 0 | 0 | 0 | 5 | 0 | 0 |
| 0 | 49 | M | 14 | 0 | 37 | 6 | 37 | 0 | 0 | 0 | 0 | 0 | 0 |
| 0 | 23 | M | 164 | 30 | 276 | 65 | 246 | 0 | 0 | 0 | 15 | 12 | 71 |
| 1 | 27 | M | 67 | 7 | 156 | 16 | 156 | 0 | 0 | 0 | 7 | 2 | 12 |
| 1 | 29 | F | 78 | 10 | 155 | 21 | 154 | 0 | 0 | 0 | 9 | 0 | 0 |
| 0 | 27 | F | 56 | 3 | 127 | 17 | 127 | 0 | 0 | 0 | 3 | 1 | 5 |
| 0 | 20 | M | 76 | 28 | 82 | 18 | 82 | 0 | 0 | 0 | 4 | 1 | 6 |
| 1 | 41 | F | 47 | 12 | 83 | 15 | 83 | 0 | 0 | 0 | 3 | 1 | 4 |
| 1 | 30 | M | 40 | 9 | 104 | 15 | 104 | 0 | 0 | 0 | 8 | 1 | 5 |
| 1 | 45 | M | 39 | 14 | 68 | 11 | 68 | 0 | 0 | 0 | 4 | 0 | 0 |
| 1 | 24 | M | 60 | 3 | 166 | 18 | 166 | 0 | 0 | 0 | 10 | 2 | 11 |
| 0 | 35 | F | 22 | 0 | 66 | 10 | 65 | 1 | 2 | 0 | 0 | 1 | 4 |
| 1 | 31 | F | 81 | 22 | 155 | 13 | 154 | 1 | 0 | 0 | 28 | 0 | 0 |
| 0 | 22 | M | 21 | 2 | 49 | 8 | 49 | 0 | 0 | 0 | 0 | 0 | 0 |
| 1 | 38 | M | 43 | 4 | 89 | 8 | 89 | 0 | 4 | 0 | 0 | 1 | 10 |
| 0 | 27 | F | 85 | 28 | 119 | 19 | 116 | 2 | 4 | 0 | 2 | 1 | 8 |
| 0 | 53 | F | 64 | 4 | 101 | 18 | 101 | 0 | 0 | 0 | 0 | 3 | 13 |
| 0 | 23 | M | 18 | 3 | 47 | 7 | 47 | 0 | 0 | 0 | 0 | 0 | 0 |
| 1 | 39 | M | 28 | 2 | 79 | 11 | 78 | 0 | 0 | 0 | 5 | 1 | 10 |
| 1 | 49 | M | 63 | 13 | 93 | 12 | 93 | 0 | 0 | 0 | 4 | 0 | 0 |
| 0 | 31 | M | 56 | 0 | 92 | 15 | 91 | 0 | 1 | 0 | 4 | 1 | 4 |
| 0 | 22 | M | 32 | 5 | 62 | 10 | 61 | 1 | 1 | 0 | 1 | 0 | 0 |
| 0 | 19 | M | 47 | 25 | 43 | 11 | 43 | 0 | 1 | 0 | 16 | 2 | 6 |
| 1 | 20 | M | 35 | 2 | 68 | 7 | 68 | 0 | 0 | 0 | 2 | 0 | 0 |
| 0 | 45 | M | 65 | 3 | 118 | 15 | 116 | 1 | 0 | 0 | 14 | 0 | 0 |
| 0 | 25 | F | 40 | 6 | 90 | 13 | 89 | 0 | 0 | 0 | 0 | 0 | 0 |
| 0 | 41 | F | 26 | 0 | 76 | 10 | 75 | 0 | 0 | 0 | 0 | 0 | 0 |
| 0 | 30 | F | 10 | 0 | 25 | 5 | 25 | 0 | 0 | 0 | 0 | 0 | 0 |
| 1 | 32 | F | 44 | 5 | 114 | 19 | 114 | 0 | 0 | 0 | 9 | 0 | 0 |
| 0 | 20 | M | 91 | 17 | 153 | 23 | 149 | 0 | 0 | 0 | 7 | 5 | 14 |
| 0 | 20 | M | 27 | 4 | 56 | 9 | 56 | 0 | 0 | 0 | 0 | 0 | 0 |
| 1 | 24 | F | 100 | 10 | 218 | 28 | 218 | 0 | 0 | 0 | 47 | 5 | 56 |
| 1 | 20 | F | 48 | 2 | 100 | 9 | 99 | 0 | 0 | 0 | 16 | 0 | 0 |
| 1 | 34 | M | 80 | 22 | 163 | 24 | 160 | 0 | 0 | 0 | 40 | 0 | 0 |
| 1 | 24 | F | 42 | 16 | 90 | 14 | 90 | 0 | 0 | 0 | 2 | 3 | 12 |
| 1 | 39 | M | 68 | 11 | 165 | 31 | 161 | 0 | 0 | 0 | 4 | 4 | 24 |
| 0 | 31 | M | 39 | 11 | 62 | 12 | 61 | 0 | 0 | 0 | 0 | 0 | 0 |
| 0 | 41 | F | 16 | 0 | 39 | 6 | 38 | 1 | 0 | 0 | 0 | 0 | 0 |
| 1 | 43 | M | 79 | 25 | 121 | 27 | 120 | 0 | 0 | 0 | 0 | 0 | 0 |
| 0 | 20 | M | 64 | 9 | 127 | 15 | 127 | 0 | 0 | 0 | 6 | 0 | 0 |
| 0 | 23 | F | 77 | 7 | 129 | 28 | 125 | 0 | 1 | 0 | 6 | 3 | 10 |
| 0 | 29 | F | 28 | 10 | 43 | 8 | 43 | 0 | 0 | 0 | 0 | 0 | 0 |
| 0 | 46 | F | 38 | 4 | 64 | 10 | 63 | 0 | 0 | 0 | 0 | 0 | 0 |
| 0 | 40 | F | 46 | 3 | 113 | 19 | 108 | 1 | 0 | 0 | 0 | 5 | 44 |
| 0 | 38 | F | 95 | 0 | 209 | 33 | 206 | 0 | 0 | 0 | 25 | 8 | 21 |
| 1 | 36 | M | 51 | 2 | 97 | 12 | 97 | 0 | 0 | 0 | 2 | 0 | 0 |
| 1 | 31 | M | 40 | 4 | 92 | 13 | 89 | 2 | 0 | 0 | 3 | 0 | 0 |
| 0 | 41 | F | 35 | 0 | 89 | 7 | 88 | 0 | 0 | 0 | 7 | 0 | 0 |
| 1 | 40 | M | 43 | 23 | 64 | 9 | 64 | 0 | 0 | 0 | 4 | 0 | 0 |
| 1 | 24 | F | 61 | 6 | 171 | 29 | 169 | 0 | 0 | 0 | 10 | 2 | 5 |
| 1 | 34 | M | 49 | 13 | 107 | 23 | 105 | 0 | 0 | 0 | 0 | 1 | 9 |
| 0 | 43 | F | 23 | 0 | 80 | 16 | 77 | 0 | 0 | 0 | 7 | 3 | 16 |
| 1 | 31 | F | 48 | 18 | 110 | 15 | 109 | 0 | 0 | 0 | 0 | 1 | 6 |
| 0 | 26 | M | 49 | 2 | 81 | 17 | 79 | 0 | 0 | 0 | 6 | 1 | 1 |
| 0 | 41 | F | 33 | 7 | 71 | 12 | 71 | 0 | 0 | 0 | 1 | 1 | 4 |
| 0 | 23 | M | 38 | 16 | 50 | 7 | 49 | 0 | 0 | 0 | 6 | 0 | 0 |
| 1 | 29 | F | 24 | 2 | 77 | 13 | 77 | 0 | 0 | 0 | 4 | 2 | 8 |
| 1 | 29 | M | 41 | 6 | 97 | 11 | 97 | 0 | 0 | 0 | 2 | 2 | 8 |
| 0 | 22 | F | 60 | 0 | 140 | 20 | 138 | 0 | 0 | 1 | 2 | 0 | 0 |
| 0 | 30 | M | 126 | 49 | 152 | 23 | 150 | 0 | 0 | 0 | 5 | 10 | 65 |
| 1 | 41 | M | 52 | 12 | 100 | 17 | 99 | 0 | 0 | 0 | 7 | 1 | 1 |
| 1 | 49 | F | 37 | 0 | 101 | 15 | 100 | 0 | 0 | 0 | 0 | 3 | 24 |
| 1 | 30 | M | 53 | 10 | 111 | 23 | 108 | 0 | 1 | 0 | 2 | 0 | 0 |
| 1 | 32 | M | 52 | 9 | 105 | 12 | 103 | 0 | 0 | 1 | 0 | 1 | 9 |
| 0 | 37 | M | 54 | 5 | 88 | 112 | 86 | 0 | 2 | 0 | 0 | 0 | 0 |
| 1 | 26 | F | 79 | 3 | 213 | 30 | 212 | 0 | 0 | 0 | 20 | 6 | 22 |
| 1 | 34 | F | 47 | 15 | 95 | 13 | 95 | 0 | 0 | 0 | 7 | 0 | 0 |
| 0 | 24 | F | 115 | 47 | 157 | 27 | 156 | 0 | 0 | 0 | 12 | 0 | 0 |
| 1 | 35 | F | 53 | 2 | 106 | 12 | 105 | 0 | 0 | 0 | 1 | 1 | 23 |
| 0 | 50 | F | 27 | 0 | 68 | 14 | 67 | 0 | 0 | 0 | 0 | 3 | 9 |
| 1 | 28 | F | 37 | 0 | 83 | 9 | 83 | 0 | 0 | 0 | 6 | 0 | 0 |
| 1 | 28 | F | 56 | 2 | 158 | 19 | 158 | 0 | 0 | 0 | 22 | 3 | 24 |
| 0 | 42 | F | 62 | 11 | 125 | 19 | 124 | 0 | 0 | 0 | 14 | 2 | 10 |
| 1 | 37 | M | 33 | 0 | 99 | 16 | 97 | 0 | 0 | 0 | 1 | 0 | 0 |
| 1 | 20 | F | 62 | 7 | 138 | 16 | 138 | 0 | 0 | 0 | 4 | 2 | 10 |
| 0 | 28 | M | 36 | 3 | 92 | 18 | 90 | 0 | 0 | 0 | 1 | 1 | 1 |
| 0 | 27 | F | 57 | 0 | 107 | 16 | 106 | 0 | 0 | 0 | 11 | 2 | 16 |
| 0 | 47 | F | 46 | 0 | 100 | 12 | 98 | 0 | 0 | 0 | 18 | 0 | 0 |
| 1 | 51 | F | 59 | 10 | 127 | 17 | 124 | 1 | 0 | 0 | 8 | 2 | 8 |
| 1 | 26 | M | 32 | 0 | 89 | 15 | 84 | 0 | 0 | 0 | 0 | 0 | 0 |
| 0 | 42 | M | 40 | 5 | 73 | 15 | 72 | 0 | 0 | 0 | 0 | 0 | 0 |
| 0 | 21 | M | 71 | 24 | 102 | 22 | 100 | 0 | 0 | 0 | 2 | 0 | 0 |
| 0 | 46 | M | 47 | 0 | 96 | 16 | 96 | 0 | 1 | 0 | 9 | 3 | 19 |
| 0 | 21 | M | 34 | 0 | 75 | 11 | 75 | 0 | 0 | 0 | 1 | 0 | 0 |
| 0 | 37 | M | 285 | 95 | 663 | 90 | 654 | 2 | 0 | 0 | 29 | 23 | 141 |
| 0 | 20 | M | 33 | 11 | 63 | 12 | 61 | 0 | 0 | 0 | 1 | 0 | 0 |
| 1 | 28 | M | 27 | 0 | 60 | 9 | 59 | 0 | 0 | 0 | 0 | 0 | 0 |
| 0 | 22 | M | 41 | 0 | 103 | 17 | 103 | 0 | 1 | 0 | 9 | 3 | 9 |
| 1 | 30 | F | 48 | 4 | 94 | 12 | 94 | 0 | 0 | 0 | 3 | 0 | 0 |
| 1 | 34 | M | 54 | 16 | 121 | 18 | 120 | 0 | 0 | 0 | 3 | 0 | 0 |
| 0 | 35 | F | 16 | 2 | 45 | 7 | 45 | 0 | 0 | 0 | 1 | 0 | 0 |
| 1 | 30 | F | 13 | 0 | 39 | 7 | 39 | 0 | 0 | 0 | 0 | 0 | 0 |
| 1 | 19 | M | 41 | 15 | 80 | 10 | 80 | 0 | 0 | 0 | 0 | 0 | 0 |
| 1 | 27 | F | 45 | 0 | 117 | 14 | 117 | 0 | 0 | 0 | 2 | 1 | 1 |
| 0 | 36 | M | 33 | 11 | 49 | 11 | 49 | 0 | 0 | 0 | 0 | 0 | 0 |
| 1 | 33 | F | 102 | 0 | 233 | 32 | 232 | 0 | 0 | 0 | 19 | 6 | 38 |
| 0 | 28 | F | 58 | 23 | 76 | 18 | 76 | 0 | 0 | 0 | 0 | 0 | 0 |
| 0 | 28 | M | 94 | 9 | 175 | 27 | 173 | 0 | 0 | 0 | 6 | 5 | 27 |
| 0 | 44 | F | 87 | 8 | 122 | 22 | 122 | 0 | 1 | 0 | 0 | 0 | 0 |
| 0 | 21 | M | 33 | 9 | 50 | 7 | 50 | 0 | 1 | 0 | 1 | 0 | 0 |
| 1 | 23 | F | 26 | 0 | 62 | 7 | 62 | 0 | 0 | 0 | 1 | 0 | 0 |
| 1 | 53 | F | 50 | 12 | 99 | 15 | 98 | 0 | 0 | 0 | 10 | 1 | 4 |
| 1 | 30 | M | 43 | 2 | 101 | 16 | 101 | 0 | 0 | 0 | 2 | 1 | 11 |
| 0 | 19 | M | 52 | 9 | 113 | 16 | 112 | 0 | 0 | 0 | 2 | 0 | 0 |
| 1 | 36 | F | 36 | 0 | 79 | 8 | 79 | 0 | 0 | 0 | 0 | 0 | 0 |
| 1 | 22 | M | 24 | 4 | 53 | 9 | 53 | 0 | 0 | 0 | 0 | 0 | 0 |
| 0 | 23 | M | 35 | 0 | 73 | 10 | 72 | 0 | 3 | 0 | 12 | 0 | 0 |
| 1 | 30 | M | 48 | 6 | 108 | 12 | 105 | 0 | 0 | 0 | 22 | 0 | 0 |
